# Strong pairwise interactions do not drive interactions in a plant leaf associated microbial community

**DOI:** 10.1093/ismeco/ycae117

**Published:** 2024-10-04

**Authors:** Franziska Höhn, Vasvi Chaudhry, Caner Bağci, Maryam Mahmoudi, Elke Klenk, Lara Berg, Paolo Stincone, Chambers C Hughes, Daniel Petras, Heike Brötz-Oesterhelt, Eric Kemen, Nadine Ziemert

**Affiliations:** Translational Genome Mining for Natural Products, Interfaculty Institute of Microbiology and Infection Medicine and Institute for Bioinformatics and Medical Informatics, University of Tübingen, 72076 Tübingen, Germany; Cluster of Excellence Controlling Microbes to Fight Infections, University of Tübingen, 72076 Tübingen, Germany; Center for Plant Molecular Biology, Interfaculty Institute of Microbiology and Infection Medicine, University of Tübingen, 72076 Tübingen, Germany; Translational Genome Mining for Natural Products, Interfaculty Institute of Microbiology and Infection Medicine and Institute for Bioinformatics and Medical Informatics, University of Tübingen, 72076 Tübingen, Germany; Center for Plant Molecular Biology, Interfaculty Institute of Microbiology and Infection Medicine, University of Tübingen, 72076 Tübingen, Germany; Cluster of Excellence Controlling Microbes to Fight Infections, University of Tübingen, 72076 Tübingen, Germany; Translational Genome Mining for Natural Products, Interfaculty Institute of Microbiology and Infection Medicine and Institute for Bioinformatics and Medical Informatics, University of Tübingen, 72076 Tübingen, Germany; Cluster of Excellence Controlling Microbes to Fight Infections, University of Tübingen, 72076 Tübingen, Germany; Cluster of Excellence Controlling Microbes to Fight Infections, University of Tübingen, 72076 Tübingen, Germany; Center for Plant Molecular Biology, Interfaculty Institute of Microbiology and Infection Medicine, University of Tübingen, 72076 Tübingen, Germany; Cluster of Excellence Controlling Microbes to Fight Infections, University of Tübingen, 72076 Tübingen, Germany; German Centre for Infection Research, Partner Site Tübingen, 72076 Tübingen, Germany; Department of Microbial Bioactive Compounds, Interfaculty Institute of Microbiology and Infection Medicine, University of Tübingen, 72076 Tübingen, Germany; Cluster of Excellence Controlling Microbes to Fight Infections, University of Tübingen, 72076 Tübingen, Germany; Department of Biochemistry, University of California Riverside, 92507 CA, Riverside, United States; Cluster of Excellence Controlling Microbes to Fight Infections, University of Tübingen, 72076 Tübingen, Germany; German Centre for Infection Research, Partner Site Tübingen, 72076 Tübingen, Germany; Department of Microbial Bioactive Compounds, Interfaculty Institute of Microbiology and Infection Medicine, University of Tübingen, 72076 Tübingen, Germany; Cluster of Excellence Controlling Microbes to Fight Infections, University of Tübingen, 72076 Tübingen, Germany; Center for Plant Molecular Biology, Interfaculty Institute of Microbiology and Infection Medicine, University of Tübingen, 72076 Tübingen, Germany; Translational Genome Mining for Natural Products, Interfaculty Institute of Microbiology and Infection Medicine and Institute for Bioinformatics and Medical Informatics, University of Tübingen, 72076 Tübingen, Germany; Cluster of Excellence Controlling Microbes to Fight Infections, University of Tübingen, 72076 Tübingen, Germany; German Centre for Infection Research, Partner Site Tübingen, 72076 Tübingen, Germany

**Keywords:** synthetic communities, plant leaf microbiomes, pyoverdines, pseudobactin, microbe–microbe interactions, correlation networks, *Arabidopsis thaliana*, secondary metabolites

## Abstract

Microbial communities that promote plant growth show promise in reducing the impacts of climate change on plant health and productivity. Understanding microbe–microbe interactions in a community context is paramount for designing effective microbial consortia that enhance plant resilience. In this study, we investigated the dynamics of a synthetic microbial community (SynCom) assembled from *Arabidopsis thaliana* leaves to elucidate factors shaping community composition and stability. We found notable disparities between *in vitro* pairwise interactions and those inferred from correlation networks *in planta*. Our findings suggested that secondary metabolites, particularly antimicrobials, might mediate interactions *in vitro*, but are not key drivers of microbial interactions in a community context. Through co-cultivation experiments, we identified the siderophore pseudobactin as a potent antimicrobial agent against several SynCom members, but its impact on community composition *in planta* was negligible. Notably, dominant SynCom members, such as *Pseudomonas koreensis, Flavobacterium pectinovorum*, and *Sporobolomyces roseus*, exhibited only positive correlations, suggesting synergism based on factors such as exopolysaccharides and biotransformation might drive community dynamics rather than competition. Two correlations between SynCom members in the co-abundance network corresponded to their pairwise *in vitro* interactions, highlighting the potential for further research and demonstrating the usefulness of correlation networks in identifying key microbe–microbe interactions. Our findings highlight the importance of considering microbiome–wide interaction studies and synthetic communities in understanding and manipulating plant microbiomes.

## Introduction

The microbiome is essential for plant survival: not only does the microbiome promote plant growth but it also increases stress tolerance to drought, salinity and iron limitation, as well as resistance to pathogens [1–4]. Strategies fighting against climate change to promote plant growth and stress tolerance are becoming more urgent. Engineering the plant microbiome using nature-derived synthetic communities, biocontrol organisms, and probiotics can be a prudent way to promote plant growth under the challenging conditions of climate change [2, 3, 5, 6]. As a proof of concept, Schmitz *et al*. used a synthetic community (SynCom) assembled from the rhizosphere of the desert plant *Indigofera* and were able to increase the salt tolerance of tomato plants [5].

For sustainable and long-term use of synthetic communities as biocontrol agents, understanding the mechanisms that shape and stabilize such communities on plants is crucial [7–9]. In this respect, not only microbe–host interactions but also overlooked microbe–microbe interactions play a role in community dynamics. Correlation networks based on co-abundance and co-occurrence of microbiome members of plants are a promising source for the detection of microbe–microbe dependencies in a community context [10–12]. Correlation network analyses of whole *Arabidopsis thaliana* microbiomes have already shown that microbe–microbe interactions are affected by environmental impacts and the plant phenotype [13–15]. Nevertheless, these factors explain only a part of the dynamics that drive microbiomes. *In vitro* pairwise interactions studies and *in situ* genome mining have revealed the enormous potential of microbiome members to produce secondary metabolites [16–19]. The identification of many genes dedicated to non-ribosomal peptides (NRPs), polyketides (PKs), ribosomally-synthesized and post-translationally–modified peptides (RiPPs), and toxins in plant microbiomes indicates a rich repertoire of potential antimicrobial agents [17, 20, 21]. Therefore, these metabolites are assumed to play a major role in plant microbiomes, however, little is known about how direct pairwise interactions of microbial members are reflected in complex microbiome interactions.

The objective of this study was to elucidate microbe–microbe interactions among core microbiome members of the *A. thaliana* leaf microbiome. Here, we refer to microbe–microbe interactions as those observed between bacterial and fungal microbiome members. As a model system, we used a SynCom from *A. thaliana* leaves based on high occurrences across multiple plant samples [13]. We investigated correlations within the community and with other members of the *A. thaliana* epiphytic microbiome based on co-abundance. We then explored correlations of these microbiome members through *in vitro* pairwise interaction studies and observed significant differences between *in vitro* relations and *in planta* correlation networks. The high number of inhibitions in pairwise interactions suggests that pairwise interactions might be driven by the production of antimicrobial secondary metabolites. This initial observation led us to question why interactions based on antimicrobial compounds are not displayed in correlation networks. By using the siderophore pseudobactin from *Pseudomonas koreensis* as an example, we showed that this strong antimicrobial agent is potent in pairwise interactions but has no effect on the SynCom composition. Strong pairwise inhibitors like *P. koreensis* and *Sporobolomyces roseus* showed >70% positive correlations *in planta* indicating that competition based on antimicrobials might play a subordinate role in the *A. thaliana* leaf microbiome. Our findings help to understand the dynamics within plant-associated microbiomes and highlight microbiome–wide correlation networks and synthetic communities as promising tools for the pre-selection of relevant microbe–microbe interactions in plant microbiome engineering efforts.

## Materials and methods

### Microbial strains and synthetic community assembly

Microbial strains for the construction of an epiphytic synthetic leaf-associated community from *A. thaliana* were isolated through a three year garden experiment (2014–2017) by Almario *et al*. In the garden experiment, plants were germinated for 10 days in the greenhouse before transferring into a field. Strain selection was based on high occurrence of operational taxonomic units (OTUs) across all plant samples from different seasons (occurrence in ≥95% of samples for fungi and ≥98% of samples for bacteria, cut off ≥10 reads per sample) as obtained by 16S ribosomal ribonucleic acid (rRNA)/ITS2 MiSeq Illumina amplicon sequencing [13]. Taxonomical classification of the SynCom members, comprising 13 bacteria and 3 fungi (Table 1), was performed through 16S rRNA and ITS2 analysis using BlastN.

**Table 1:** SynCom members characterized by 16S rRNA and ITS2 similarity (BlastN).

Closest type species match	Short name used in this study	Closest type strain match	% identity type species	Genome NCBI accession number
*Aeromicrobium fastidiosum*	*A. fastidiosum*	DSM 10552(T)	99.30	JAMKCA000000000
*Arthrobacter humicola*	*A. humicola*	KV-653(T)	100	JAFKON000000000
*Bacillus altitudinis*	*B. altitudinis*	41KF2b(T)	100	JAFKOO000000000
*Dioszegia hungarica*	*D. hungarica*	CBS 4214	100	JAMRJJ000000000
*Flavobacterium pectinovorum*	*F. pectinovorum*	DSM 6368(T)	98.61	JAFEVZ000000000
*Frigoribacterium faeni*	*F. faeni*	801(T)	99.82	JAIXNG000000000
*Massilia aurea*	*M. aurea*	AP13T	100	JBFMMP000000000
*Methylobacterium goesingense*	*M. goesingense*	iEII3(T)	99.43	JAFGZG000000000
*Microbacterium proteolyticum*	*M. proteolyticum*	RZ36(T)	99.29	JAFKOM000000000
*Nocardioides cavernae*	*N. cavernae*	YIM A1136(T)	99.23	JALQCQ000000000
*Paenibacillus amylolyticus*	*P. amylolyticus*	NBRC 15957(T)	99.49	JAMGVX000000000
*Pseudomonas koreensis*	*P. koreensis*	Ps 9–14(T)	100	JAFEVY000000000
*Rhizobium skierniewicense*	*R. skierniewicense*	Ch11(T)	99.64	JAFFPP000000000
*Rhodotorula kratochvilovae*	*R. kratochvilovae*	CBS 7436	99.82	JAFEUJ000000000
*Sphingomonas faeni*	*S. faeni*	MA-olki(T)	99.50	JALPNF000000000
*Sporobolomyces roseus*	*S. roseus*	CBS 486	99.29	JAFEUI000000000

### Culture media and conditions

Bacterial strains were pre-cultured on nutrient agar (NA), (BD, USA) or in nutrient broth (NB), (BD, USA) for 48 h. Fungi were pre-cultured on potato dextrose agar (PDA), (Carl Roth, Germany) potato dextrose broth (PDB), (Carl Roth, Germany) for 48 h. For cross-streaking experiments with *P. koreensis* wild-type (WT) and the *P. koreensis* pseudobactin deficient mutant (*ΔpvdI/J*), siderophore promotive F-base agar, which is used for the identification of fluorescent Pseudomonades (Merck, Germany), was used. Growth measurements were performed in MM9 minimal medium [22] and enriched MM9 medium, where MM9 was mixed 1:5 with NB, for bacteria and in PDB for fungi (Table S1 and S2). MM9/7 agar was used to culture the SynCom for 5 days for amplicon sequencing (Table S3). Therefore, MM9 medium was modified to a more defined agar, by exchanging casamino acids with a defined amino acid solution and the addition of agar-agar. This was done to mimic amino acids present on the leaf surface [23]. All cultures were incubated at 22°C and liquid cultures were shaken at 120 rpm.

### Sterile plants and plant spraying

Seeds of *A. thaliana* Ws-0 (Wassilewskija) were sterilized over night with chlorine gas. Therefore, seeds were incubated in presence of 4 mL concentrated hydrochloric acid in 100 ml sodium hypo chloride and 35 mbar vacuum for 6–8 h. Sterile seeds were immediately sown on 0.5 × MS agar (1.5 mM CaCl_2_, 0.63 mM KH_2_PO_4_, 9.4 mM KNO_3_, 0.75 mM MgSO_4_, 10.3 mM NH_4_NO_3_, 0.055 μM CoCl_2_ × 6 H_2_O, 0.05 μM CuSO_4_ × 5 H_2_O, 50.00 μM FeNaEDTA, 50.15 μM H_3_BO_3_, 2.5 μM KI, 50 μM MnSO_4_ × H_2_O, 0.52 μM Na_2_MoO_4_ × 2 H_2_O, 15 μM ZnSO_4_ × 7 H_2_O, 8 g/L agar-agar). Plates were sealed with leukopor (BSN medical GmbH, Germany) and grown in a short day chamber (8 h light, 16 h dark, 21°C, 50% humidity) for 1–2 weeks. Seedlings were picked and placed in 12-well plates containing 0.5 MS agar. Plants were further incubated for 2 weeks in a short-day chamber.

For spraying, each SynCom strain was pre-cultured in 20 mL of liquid medium. Cultures were harvested after 48 h of incubation through centrifugation at 7.000 rpm for 5 min. Afterwards, cells were washed twice and resuspended in 10 mL of 10 mM MgCl_2_. For each strain, the optical density at wavelength 600 nm (OD_600_) was measured and the cultures were diluted to OD_600_ = 0.2. Equal volumes of each strain dilution were combined to form the different SynCom groups used for amplicon sequencing. The mixtures, augmented with 0.02% silwet 700 for finer droplet distribution, were sprayed onto sterile 3-week-old *A. thaliana* plants using an airbrush system with 2 mbar pressure applied through 2 brushes. Following inoculation plants were incubated in short-day chambers (8 h light, 16 h dark) at 22°C.

### Correlation network analysis

To investigate the abundance and connectivity of SynCom members in the epiphytic leaf microbiome of wild *A. thaliana* plants, correlation networks were constructed. Since the study from which the SynCom was assembled [13] was based on a common garden experiment, we used a different dataset based on samples from wild *A. thaliana* plants to calculate correlation networks. The networks were based on OTU tables generated in the study of Mahmoudi *et al*. [24]. There, the data was collected from 351 wild *A. thaliana* plants sampled around Tübingen. Plants were collected for 5 years (2014–2019) in fall and spring, the deoxyribonucleic acid (DNA) was isolated and 16S/ITS2 amplicon sequencing was performed. In brief, bacterial and eukaryotic (fungal and non-fungal) OTU tables were filtered to retain only those OTUs present in at least 5 samples with >10 reads. The OTU tables were used to calculate SparCC correlations [25] (with default parameters) in the FastSpar platform [26]. Permuted *P*-values for each correlation were derived from 1.000 bootstraps datasets. Only correlations with *P* ≤ .001 were kept for further analysis. The preparation of OTU tables from the raw data followed the workflow of Mahmoudi *et al*. [24]*,* afterwards correlations were calculated as shown in the workflow stored at Zenodo repository (strong pairwise interactions do not drive interactions in a plant leaf associated microbial community; https://zenodo.org/records/12795858). 16S and ITS2 regions of all SynCom members were aligned by BlastN to the most common 16S and ITS2 regions of OTUs after correlation. The closest BlastN match for each SynCom member was assigned as representative (Table 2). Cytoscape (version 3.10.0) [27] was used for visualization of interactions of epiphytic leaf microbiome members and SynCom microorganisms on genus level.

**Table 2:** Correlation network OTU annotation by 16S rRNA/ITS2 BlastN against SynCom members. The most common 16S rRNA/ITS2 sequence of each OTU was blasted against 16S RNA/ITS2 regions of SynCom members to identify the closest related nodes in the correlation network for each SynCom members.

Node name (family or genus)	OTU number	Related SynCom strain	BlastN similarity of 16S rRNA/ITS2 in %
*Verrucariaceae*	OTU00184	*S. roseus*	100
*Arthrobacter*	OTU000363	*A. humicola*	99.50
*Microbacterium*	OTU000360	*M. proteolyticum*	100
*Flavobacterium*	OTU000009	*F. pectinovorum*	99.20
*Massilia*	OTU000172	*M. aurea*	98.90
*Pseudomonas*	OTU000144	*P. koreensis*	99.50
*Aeromicrobium*	OTU000030	*A. fastidiosum*	98.90
*Methylobacterium*	OTU000003	*M. goesingense*	98.90
*Allorhizobium*	OTU000014	*R. skierniewicense*	99.70
*Sphingomonas*	OTU000002	*S. faeni*	100
*Nocardioides*	OTU000071	*Navicula cavernae*	99.70
*Paenibacillus*	OTU001595	*P. amylolyticus*	100

### Cross-streaking experiments

Pairwise interactions of SynCom members were observed on NB and PDA. Previous experiments determine the media as best fit for an equally growth of all SynCom members. Solid pre-cultures were taken with cotton swaps, resuspended in 10 mM MgCl_2_ and diluted to OD_600_ 1.0. Using a fresh cotton swap, test strains were streaked out on NA/PDA agar plates. Once the test strain was dry, all SynCom members were streaked crosswise onto the test strain. Inhibiting interactions were visually observed after 48 h incubation by the production of inhibition zones. Therefore, cross-streakings are qualitative observations of pairwise interactions. Promoting interactions were observed by higher growth in contact zones (Fig. S1). Cross-streaking experiments to test the effect of pseudobactin on all SynCom members were performed on F-base agar for 48 h using *P. koreensis* WT and the *∆pvdI/J* mutant. Cross-streaking experiments on NA/PDA were repeated three times. Cross-streakings on F-base agar were repeated two times.

### Genome mining

Genomes of SynCom members (references see Table 1) were analyzed by AntiSMASH 7 [28] for the presence of biosynthetic gene clusters (BGCs) of secondary metabolites. Similarities to known compounds were further investigated by MIBiG [29] comparison and BlastN/BlastP analysis.

### Pseudobactin identification and purification

*P. koreensis* cultivated in 1 L MM9 medium for 48 h at RT and 100 rpm shaking was used for high-performance liquid chromatography-mass spectrometry (HPLC-MS) and nuclear magnetic resonance (NMR) analysis. Cells were harvested by centrifugation at 8.000 rpm for 5 min and supernatant was collected. The supernatant was tested for the presence of pseudobactin under ultraviolet (UV) light (365 nm) and by HPLC-MS analysis. HPLC-MS measurements were performed on an Agilent 1260 Infinity (Agilent technology, USA) using a Kinetex 5 μm 100 Å, 100 × 4.6 mm C18 column and a single-quadrupole G6125B MSD in positive ion mode. Analytical HPLC was performed by using the following parameters: 5 μL injection; solvent A: H_2_O [0.1% trifluoroacetic acid (TFA)]; B: acetonitrile (0.1% TFA); gradient eluent: 10%–100% B over 10 min, 100% B for 2 min, and requilibration to initial conditions >3 min; flow rate: 1.0 mL/min; UV detection: 254 nm; retention time: 1.2 min; pseudomolecular ion: *m/z* [M + H] + = 989.4. For the purification of pseudobactin, the supernatant was loaded onto a C18 cartridge (Supleco, USA). The cartridge was washed with 100% water (0.1% TFA), and pseudobactin was eluted with 10% acetonitrile (0.1% TFA). The fraction was dried using a rotary evaporator and lyocell vacuum evaporator. A total of 20 mg of the dry sample was resuspended in methanol (1 mL), and preparative HPLC was performed by using the following parameters: solvent A: water (0.1% TFA); solvent B: methanol (0.1% TFA); isocratic eluent: 15% B; flow rate: 10 mL/min; UV detection: 254 nm; retention time: 20.5 min. Fractions containing pseudobactin were collected, dried, and analyzed by NMR spectroscopy. ^1^H NMR and 2D spectra were recorded at 700 MHz in D_2_O (4.79 ppm). ^13^C NMR spectra were recorded at 175 MHz in D_2_O (not referenced).

### Deletion mutant creation

For the investigation of interaction between SynCom members and pseudobactin, a pseudobactin deletion mutant of *P. koreensis* was constructed. For the deletion, genes *pvdI* and *pvdJ* were chosen [30, 31]. The deletion of the genes was performed as described by Huang *et al* [32]. In short, the deleted gene region was cloned in the vector plasmid pEX18Gm by Gibson assembly. The vector containing the deleted region was transferred into *P. koreensis* by conjugation with *E. coli* S17-λ as donor. The genes were introduced into the *P. koreensis* genome through a single crossover and the plasmid backbone containing the WT copy of gene region was subsequently eliminated under selection pressure on antibiotic plates. The success for the deletion was confirmed by polymerase chain reaction (PCR) of the deletion region, HPLC-MS analysis, and UV measurement. All primers for the construction and verification of the deletion are shown in supplementary material (Table S4).

### Pseudobactin interaction studies

Feeding experiments were performed by growing SynCom members in medium supplemented with *P. koreensis* WT and *∆pvdI/J* mutant supernatant. Therefore, the supernatant of 1 L *P. koreensis* WT and mutant was collected by culturing the strains in MM9 medium. Cells were harvested at 8.000 rpm for 5 min and supernatant was sterilized by filtering (0.2 μm pore size). The optimal growth media were developed as MM9 and enriched MM9 medium supplemented with the sterile supernatant of *P. koreensis* WT or ∆*pvdI/J* mutant. For a detailed recipe, see supplements (Table S1 and S2). Since MM9 is a minimal medium, some SynCom strains were not able to grow under these conditions. For these organisms, enriched MM9 medium was used with minimal additions of NB or PDA medium. For the growth curves, each SynCom strain was pre-cultured, washed, and diluted to OD_600_ = 0.2 with MM9 or enriched MM9 medium. 1 mL of each dilution was added into one well of a 24-well plate. Experiments were performed in triplicates. Plates were incubated at 22°C and 100 rpm shaking, and OD_600_ was measured after T0 = 0 h; T1 = 16 h; T2 = 18 h; T3 = 20 h; T4 = 22 h; T5 = 24 h; T6 = 40 h; T7 = 42 h with a TECAN 2000 (Tecan, Switzerland) device. For additional growth curves with *Arthrobacter humicola*, 48-well plates and 800 μl total volume were used. Strain was diluted to OD_600_ = 0.2 in the well. For complementing the inhibiting effect of pseudobactin, *A. humicola* was further investigated in WT + FeSO_4_ MM9 medium. For complementing the *∆pvdI/J* mutant, *A. humicola* was cultivated in *pvdI/J* + pure pseudobactin MM9 medium (Table S1 and S2). Growth curves for all SynCom members were prepared in two independent experiments.

Additionally, cross-streaking experiments of all SynCom members against *P. koreensis* WT and *∆pvdI/J* were performed on F-base agar to investigate inhibitions caused by the production of pseudobactin. See materials and methods part—cross-streaking experiment—for more details.

### Amplicon sequencing

#### Sample preparation

For investigating the relative abundance of SynCom members *in vitro*, amplicon sequencing from the microorganisms grown on MM9/7 agar plates was performed. In detail, each SynCom member was pre-cultured in 20 mL liquid medium and harvested after 48 h incubation by centrifugation at 7.000 rpm for 5 min. Cells were washed twice using 10 mL of 10 mM MgCl_2_ and resuspended in MgCl_2_. OD_600_ = 1.0 was adjusted, and strains were mixed in equal volumes. In total, 1 mL mixture was streaked on MM9/7 agar plates and incubated for 5 days at 22°C. After incubation, cells were scratched off the agar in bead filled tubes (MP fastDNA spin kit) and immediately frozen in liquid nitrogen. Samples were prepared in three independent experiments.

For investigating the relative composition of the SynCom *in planta*, amplicon sequencing from plants was performed. Therefore, SynCom WT, SynCom mutant and SynCom pseudobactin sprayed plants were picked in bead filled tubes (MP fastDNA spin kit) after 5 and 9 days of incubation. Tubes were immediately frozen in liquid nitrogen and plants were crushed at –30°C using a Precellyse device (Bertin, France) (2 × 20 s, 6.400 rpm). Samples were prepared in three independent experiments. In each experiment, each group was sampled in biological triplicates including two pooled technical replicates. In total 108 plants were sampled.

#### Deoxyribonucleic acid isolation

DNA for amplicon sequencing was isolated using the MP fastDNA spin (MP Biomedicals, Germany) kit for soil according to manufacturer’s instructions. DNA was eluted in 75 μL elution buffer. Concentration was measured by nanodrop.

#### Library preparation

The library preparation was done following the studies of Agler *et al*. and Mayer *et al*. [15, 33]. Shortly, DNA was used to amplify the 16S rRNA region of bacteria and the ITS2 region of fungi by PCR. A second PCR was used to introduce custom-designed, single indexed Illumina sequencing adapters to each sample. The primers used contained blocking regions to limit the amplification of plant chloroplast DNA as described in the study of Mayer *et al*. All libraries were pooled in equimolar concentrations and sent to NCCT/University of Tübingen for MiSeq Illumina sequencing (300 cycles). Primers and Illumina adapters used in this study can be found in the article of Agler *et al*. [15]

#### Data analysis

Quality control and trimming of raw reads were performed using fastp (v0.23.4) [34] with default parameters. The demultiplexed raw reads were denoised using DADA2 [35] truncating left and right reads at the 250th and 200th positions, respectively, based on a manual inspection of quality scores. The taxonomic analysis of the amplicon sequence variants (ASV) was carried out using QIIME2 with sklearn classifier [36] against the SILVA database (v138, 99%) [37] for bacterial sequencing runs and the UNITE database (v0.9, 99%) [38] for fungal sequencing runs. The raw read counts for ASVs were exported from QIIME artifacts and used in further analysis. Any taxa that have <1% cumulative mean relative abundance were grouped under the category “Other” in the figures. ASVs assigned to *Chloroplast sp.* and *Penicillium sp.* were excluded from the analysis. Significances between experimental conditions and taxa were tested by a Kruskal test, followed by pairwise Wilcoxin test. *P*-values were corrected using Bonferroni. Additionally, a *t*-test was performed to determine differences in organismic abundances (*P*-value ≤.05%).

## Results

### Synthetic community members are mainly positively correlated with the epiphytic microbiome

The SynCom was assembled in garden experiments from *A. thaliana* leaves based on the occurrence of the taxonomic unit in plant samples. Bacteria present in >98% and fungi present in >95% of plant samples during different seasons were collected [13].

**Figure 1:**
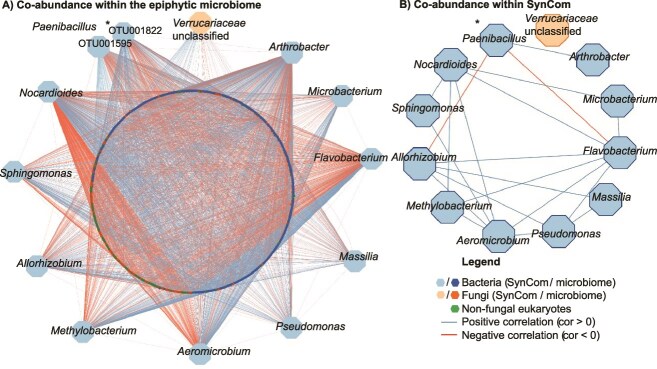
Correlation of SynCom members within the *A. thaliana* epiphytic microbiome based on co-abundance. (A) Each node represents an OTU calculated by 16S rRNA/ITS2 Illumina amplicon sequencing. OTUs were identified on genus level. Based on 16S rRNA/ITS2 similarity (BlastN) to SynCom members, closest related OTUs were presented. The network shows correlations with *P* ≤ .001. ^*^OTU001822 and OTU001595 showed the same BlastN similarity to *P. Amylolyticus* and were both kept in the network. (B) SynCom-related OTUs and edges were extracted from network a. ^*^OTU001822 showed less, but same correlations as OTU001595 and therefore was replaced.

Since the SynCom was assembled from *A. thaliana* grown on an experimental field, we fist aimed to analyze the abundance and connectivity of the SynCom members in naturally grown *A. thaliana* plants. Therefore, we correlated the co-abundance of OTUs with closest similarity to the SynCom members in a new dataset from wild *A. thaliana* samples. The data was generated in the study from Mahmoudi *et al*. [24]. The authors therefore sampled plants >5 years at different spots around Tübingen, isolated DNA and performed 16S / ITS2 amplicon sequencing. The positive and negative correlations shown in the network were based on co-abundance in all field samples. OTUs showing no significant abundance dependencies (*P* > .001) are not shown in the network. Alignment of the most common sequence of each OTU to 16S rRNA and ITS2 sequences of SynCom members facilitated the identification of OTUs closest related SynCom members (Table 2). The generated network (*P* ≤ .001) allowed the identification of positive (blue) and negative (red) correlations between SynCom members and the *A. thaliana* epiphytic microbiome (Fig. 1A).

Overall, SynCom organisms showed a total of 4.116 correlations with OTUs from the phyllosphere microbiome. Among these correlations, the majority (59.5%) was positive, while 40.5% were negative. Four SynCom members, namely *Bacillus altitudinis, Frigoribacterium faeni*, *Dioszegia hungarica*, and *Rhodotorula kratchovilovae*, remained uncorrelated within the network due to their infrequent occurrence and/or low read counts (Table S5). Among the represented SynCom strains, nine exhibited notably high positive correlations (>60% OTUs positively correlated) within the microbiome. Noteworthy exceptions included *A. humicola* (54.6% OTUs positively correlated), which also displayed the highest total number of correlations (811 interactions), along with *Flavobacterium pectinovorum* (46.6% OTUs positively correlated) and *Nocardioides cavernae* (44.1% OTUs positively correlated). The highest positive correlation was recorded for *Sporobolomyces roseus* (82.8%), closely followed by *P. koreensis* (74.0%; Fig. S3). Analysis of connections between SynCom members derived from the microbiome network revealed predominantly positive associations, comprising 20 correlations, with only two relationships exhibiting negative ratios (Fig. 1B). Particularly noteworthy were the highly positive correlations observed for *F. pectinovorum* and *Sphingomonas faeni*, both displaying positive linkages with six other SynCom members. Notably, *Paenibacillus amylolyticus* emerged as the sole OTU exhibiting negative correlations with two other SynCom members (*F. pectinovorum* and *Rhizobium skierniewicense*). In summary, the SynCom members exhibited predominantly positive correlations within the microbiome, both with other microbiome constituents and among themselves.

### Pairwise interactions do not explain relations from correlation networks

**Figure 2:**
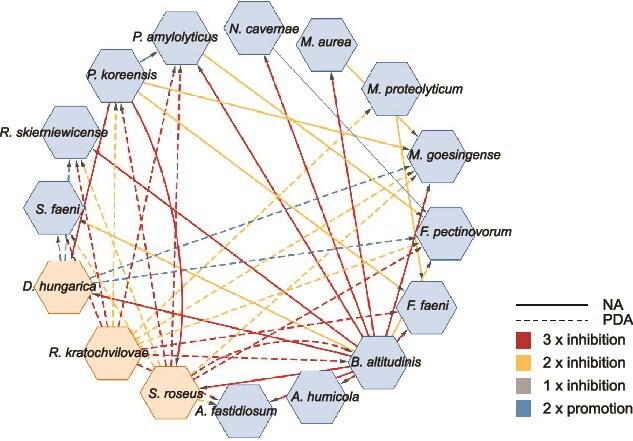
Pairwise interactions of SynCom members *in vitro*. Strains were grown on optimal growth medium for 3 days at 22°C (NA for bacteria, PDA for fungi). Contact zones were visually assessed for growth-promoting or inhibiting interactions (for examples, see Fig. S1).

We further investigated whether relations shown in the correlation network (Fig. 1B) could be followed up in pairwise interactions *in vitro*. Therefore, we compared the network data with pairwise interactions observed between SynCom members in cross-streaking experiments on agar plates. Each organism within the SynCom was subjected to cross-streaking against every other member, resulting in a total of 256 tested interactions (Fig. 2).

While most strains exhibited neutral co-existence, six reproducible growth-promoting interactions were identified among SynCom members across two repetitive experiments. Notably, the yeast *D. hungarica* promoted the growth of four SynCom members (*Methylobacterium goesingense*, *F. pectinovorum*, *S. faeni*, and *R. skierniewicense*) on their optimal growth agar (potato dextrose agar, PDA). Only one positive interaction was observed between the bacteria *F. pectinovorum* and *N. cavernae*, aligning with the positive correlation observed in the correlation network. The pairwise interaction network predominantly featured negative interactions (Fig. 2). A total of 37 inhibitory relationships were identified, exhibiting 59% reproducibility across three independent experiments and 41% occurring in two of the three repetitions. Among these, 21 interactions originated from bacteria, and 22 from fungi. Notably, *B. altitudinis* emerged as the most potent bacterial inhibitor within the SynCom, displaying inhibitory effects against 12 SynCom strains in pairwise assessment. However, despite its strong inhibitory activity, *B. altitudinis* was not represented in the correlation networks due to low OTU reads for the strain (Table S5).

Another prominent inhibitor in pairwise interactions was *P. koreensis*, which inhibited four other SynCom members (*M. goesingense, F. faeni, D. hungarica, S. roseus*) reproducibly. Interestingly, *P. koreensis* exhibited solely positive correlations with SynCom members within the correlation network.

The most susceptible strain was *M. goesingense*, which displayed sensitivity to five partners in the cross-streaking experiment. However, the strain showed a high number of positive relationships with the SynCom and other epiphytic microorganisms in the correlation network.

Among the fungi, *R. kratochvilovae* (11) and *S. roseus* (10) exhibited the highest number of inhibitory interactions. Interestingly, the inhibitory potential of both fungi was only evident when grown on PDA. *R. kratochvilovae* and *S. roseus* consistently restricted the growth of potent bacterial inhibitors such as *B. altitudinis* and *P. koreensis* on this medium. Conversely, *B. altitudinis* and *P. koreensis* inhibited *S. roseus* on nutrient agar (NA). These findings underscore the significant impact of optimal nutrient availability on the SynCom’s pairwise interactions. Collectively, contrary to the correlation network analysis, pairwise interactions unveiled a substantial repertoire of inhibitory interactions among SynCom members. This prompted us to investigate the reasons behind this discrepancy.

### The synthetic community encodes a variety of secondary metabolite gene clusters

**Figure 3:**
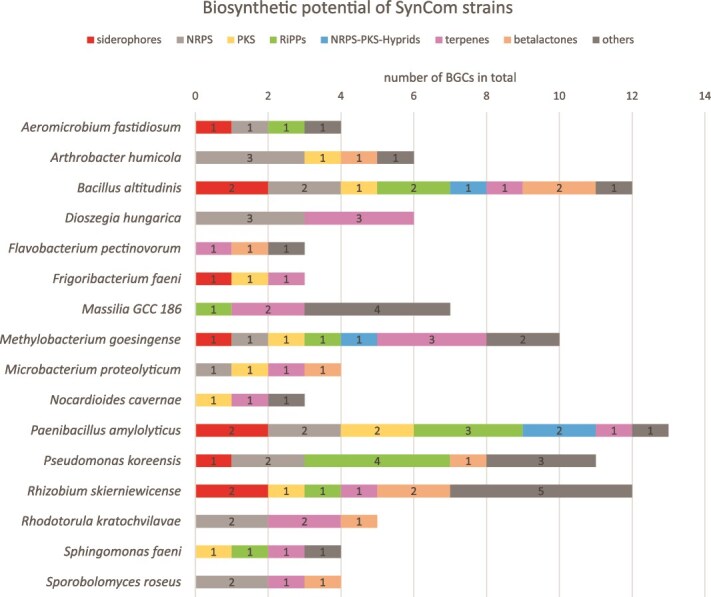
Potential of SynCom strains to produce secondary metabolites. The potential to produce secondary metabolites is based on the presence of BGCs as revealed by AntiSMASH 7 analysis.

Antagonistic microbe–microbe interactions are a common phenomenon in pairwise interactions of members from the *A. thaliana* leaf microbiome [17, 39]. Most inhibitions are attributed to the vast repertoire of antimicrobial compounds synthesized by a diversity of biosynthetic enzyme classes [16, 17]. To investigate whether the observed inhibitory pairwise interactions are caused by antimicrobial compounds, we analyzed the potential of each SynCom member to produce secondary metabolites. Therefore, we utilized AntiSMASH, a tool for predicting BGCs. Figure 3 illustrates the abundance of BGCs among SynCom strains, totaling 103 gene clusters. *P. amylolyticus* encodes the highest number of BGCs (13), followed by *B. altitudinis* (12), *R. skierniewicense* (11), *P. koreensis* (10), and *M. goesingense* (10). Interestingly, these organisms, except for *M. goesingense,* exhibit significant potential for antimicrobial compound production, based on the presence of RiPP, PKS, NRPS, and hybrid gene clusters. Furthermore, examination of gene clusters from the inhibitor strains in pairwise interactions revealed similarities to BGCs encoding known antimicrobials. For instance, *B. altitudinis* possesses BGCs closely resembling those encoding antimicrobials such as bacilysin (100% similarity), surfactin (85% similarity), and bacillibactin (53% similarity). *P. koreensis* exhibits genes associated with the production of the siderophore pseudobactin from the pyoverdine class, while a 100% similarity to the BGC of polymyxin B was predicted for one NRPS gene cluster of *P. amylolyticus*. In contrast, strains showing higher sensitivity in pairwise interactions, such as *F. pectinovorum and F. faeni*, lack NRPS and PKS gene clusters, and are characterized by the presence of terpene and beta-lactone BGCs (Table S6). The two strong inhibitory fungi *R. kratochvilovae* and *S. roseus* carry a low number of BGCs (4) compared to their bacterial equivalents. Both strains contain two NRPS gene clusters with no similarity to known BGCs. Notably, *D. hungarica* carrying 3 NRPS BGCs shows no inhibition in pairwise interactions.

### Pairwise inhibitors are not inherently dominant strains in the synthetic community *in vitro*

**Figure 4:**
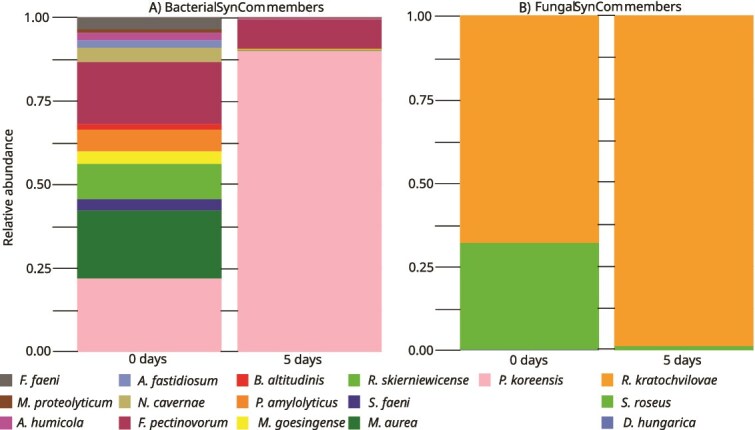
SynCom composition *in vitro* based on the relative abundance. The SynCom composition *in vitro* was calculated as relative abundance of each strain by 16S rRNA/ITS2 MiSeq Illumina amplicon sequencing from MM9/7 agar at inoculation after 0 and 5 days incubation at 22°C. (A) Histograms show the relative abundance of bacterial SynCom members calculated by amplicon sequencing using 16S rRNA specific primers. (B) Histograms show the relative abundance of fungal SynCom members calculated by amplicon sequencing using ITS2-specific primers.

As a next step, we wanted to investigate whether pairwise interactions play a role in shaping the SynCom *in vitro*. We posited that inhibitor strains might exhibit a colonization advantage within the community by producing antimicrobial compounds, leading to their dominant abundance. To investigate this, amplicon sequencing of the entire SynCom cultivated together on minimal agar was conducted (Fig. 4). Therefore, equal volumes of OD_600_ = 1.0 mixtures of each strain were mixed. For the experiment, MM9/7 minimal agar was chosen to mimic the limited nutrient bioavailability on plant leaf surfaces [40, 41]. Following a 5-day incubation period, *P. koreensis* emerged as the most prevalent bacterium within the SynCom, with an 89% relative abundance (Fig. 4). Notably, the relative abundance of *P. koreensis* increased fourfold over the incubation period (Fig. S5). Regarding fungi, *R. kratochvilovae* showed the highest relative abundance at 70% (Fig. 4). Interestingly, both *R. kratochvilovae* and *P. koreensis*, recognized as potent inhibitor strains in pairwise interactions, displayed the highest abundance within the SynCom on the plate. In contrast, *B. altitudinis*, able to inhibit 14 SynCom strains in the preceding experiment, showed a ~ 19-fold reduction in abundance over the incubation period, resulting in a total relative abundance of <0.5% (Fig. S4). Strains susceptible to inhibition, such as *F. pectinovorum* and *R. skierniewicense*, were relatively abundant compared to the main bacterial inhibitor strain, *B. altitudinis*. Furthermore, besides *P. koreensis*, *F. pectinovorum* was the only bacterial strain with high abundance after the incubation period. Although the strong inhibitors *P. koreensis* and *R. kratochvilovae* were dominant colonizers, the low abundance of other strong inhibitor strains like *B. altitudinis* and *S. roseus* indicates that inhibitors do not inherently have a colonization advantage within the SynCom *in vitro.* Therefore, pairwise interactions are not necessarily reflected by the composition of the SynCom on agar plates.

### Pseudobactin drives inhibitory pairwise interactions of *Pseudomonas koreensis*

Next, we aimed to answer the question why strong pairwise interactions are not reflected in the correlation networks. Therefore, we aimed to identify the mechanism behind a specific inhibitory pairwise interaction, track it through subsequent studies from pairwise interactions to co-cultures with the entire SynCom, and finally, examine it *in planta*.

**Figure 5:**
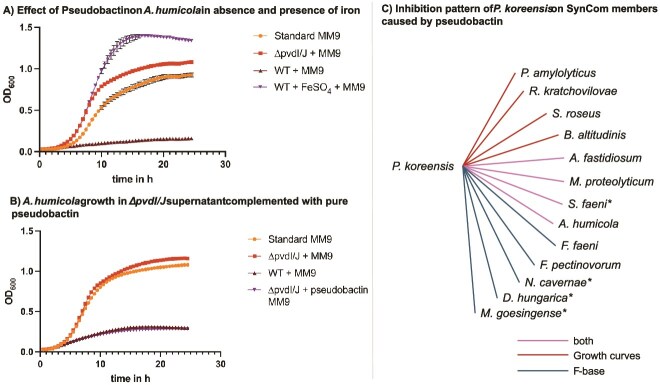
*In vitro* interaction of pseudobactin with single SynCom member’s growth curves of *A. humicola* were measured automatically in a TECAN 2000 device as OD600 in 1 h intervals at 22°C and 200 rpm shaking. (A) Growth curve of *A. humicola* in MM9 medium enriched with sterile supernatant of *P. koreensis* WT and *P. koreensis ∆pvdI/J* mutant. As a control for iron limitation, WT supernatant was complemented with FeSO_4_. Growth curves were prepared in triplicates and reproduced once. (B) Repetition of growth curve of *A. humicola* with complementation of *∆pvdI/J* mutant supernatant with pure pseudobactin. (C) Summary of inhibitions of pseudobactin on SynCom members in growth curves and cross-streaking experiment on F-base agar. Cross-streakings on F-base agar were prepared in two independent experiments. ^*^Strains showing inhibition zones in contact with both WT and mutant *P. Koreensis* but significantly bigger inhibition zones in contact with WT.

Due to its high abundance in the SynCom on the plate, its ability to inhibit individual SynCom members and its opposing interactions in the correlation network, *P. koreensis* was chosen for further investigations. Previous studies showed that a pyoverdine siderophore with antimicrobial activity contributes to shaping the root microbiome of *A. thaliana*, suggesting its importance in microbial communities [16]. Since a BGC encoding a pyoverdine was detected in the *P. koreensis* genome, we investigated its role in the leaf associated SynCom. The fluorescent compound was isolated, and its structure confirmed by NMR as pseudobactin, a member of the pyoverdine siderophore class (Figs S6–S13). The successful creation of a deletion mutant was verified by HPLC-MS (Fig. S14). Growing strains in the presence or absence of pseudobactin showed inhibitions of growth for eight SynCom members (Fig. 5C; growth curves of SynCom strains: Fig. S15). *A. humicola* was significantly inhibited by pseudobactin. The addition iron to *A. humicola* cultures abolished the inhibiting effect (Fig. 5A), leading to normal growth in pseudobactin-containing medium. The restoring of the inhibition by the addition of iron indicates that *P. koreensis* inhibits SynCom members indirectly by the chelation of iron. The addition of purified pseudobactin to the supernatant of the pseudobactin mutant strain reestablished the inhibiting effect (Fig. 5B).

The inhibitory effects of *P. koreensis* on four SynCom members can therefore be explained by the production of pseudobactin*.* Notably, three strains, namely *R. kratochvilovae, B. altitudinis*, and *A. humicola*, which were not initially inhibited in pairwise interactions, demonstrated susceptibility when exposed to the siderophore in growth curves. The fife SynCom strains (*N. cavernae*, *M. goesingense*, *D. hungarica*, *Massilia aurea*, and *R. skierniewicense)* exhibited instability in growth when cultivated in minimal medium. Consequently, it was not possible to ascertain their growth rate in the presence of pseudobactin, precluding the formulation of definitive statements regarding their response to this antimicrobial agent. To investigate the effect of pseudobactin under condition, where these strains were able to grow, cross-streaking experiments on siderophore production agar (f-base agar) were performed (Fig. 5C). Therefore, all SynCom members were streaked against *P. koreensis* WT and the pseudobactin mutant. Although, *M. goesingense*, *N. cavernae*, and *D. hungarica* were sensitive against both, WT and mutant *P. koreensis*, they showed larger zones of inhibition in contact with the WT, indicating some sensitivity to pseudobactin but also other compounds produced by *P*. *koreensis. M. aurea and R. skierniewicense* were resistant against pseudobactin on f-base agar. Interestingly, the second most abundant bacterium, *F. pectinovorum*, confirmed its resistance to *P. koreensis* WT observed in pairwise interactions in the growth curves but showed susceptibility to pseudobactin on F-base agar. Fife SynCom members (Fig. 5C, red) were susceptible to pseudobactin in growth curves, but not on f-base agar. In summary, pseudobactin is a compound of *P. koreensis* showing antimicrobial activity in pairwise interaction studies on siderophore promotive agar (F-base) and in minimal medium.

### Pseudobactin interactions show no effect in a community context *in planta*

**Figure 6:**
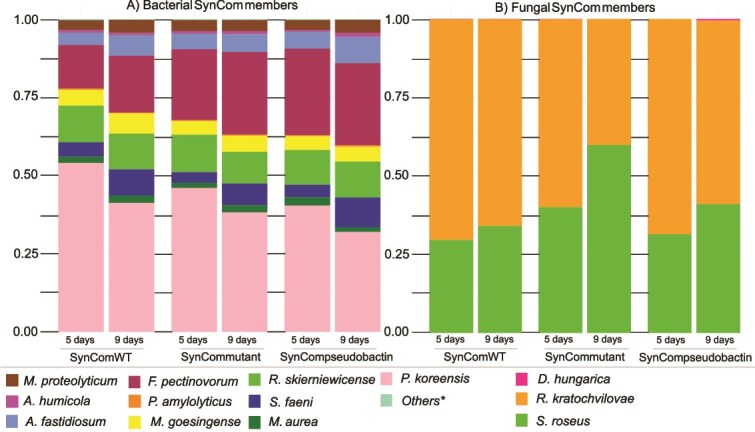
The effect of pseudobactin on the SynCom composition *in planta.* The composition of the SynCom is based on the relative abundance of each member calculated from 16S rRNA/ITS2 MiSeq Illumina amplicon data. Three-week-old plants were sprayed with 0.2 OD_600_ SynCom mixture and incubated at 22°C in a short light chamber. The sampling was done at two time points (5 and 9 days after spraying). (A) Histograms show the relative abundance of bacterial SynCom members calculated by amplicon sequencing using 16S rRNA-specific primers. (B) Histograms show the relative abundance of fungal SynCom members (fungi) calculated by amplicon sequencing using ITS2-specific primers. ^*^Others include remaining SynCom members with relative abundances <1% (*B. altitudinis*, *F. faeni*, and *N. cavernae).*

Since the pseudobactin-based inhibitory interactions of *P. koreensis* are not reflected in correlation networks, we further wanted to investigate which role pseudobactin plays within the SynCom *in planta.* Given the known influence of pyoverdines on microbiome composition, we assessed the contribution of pseudobactin by applying three different SynCom preparations to sterile *A. thaliana* plants through plant spraying: the wild type SynCom containing *P. koreensis* (SynCom WT), the SynCom with the *P. koreensis* pseudobactin mutant (SynCom mutant), and the SynCom mutant supplemented with pure pseudobactin (SynCom pseudobactin). Using amplicon sequencing, we determined SynCom member abundance on plants (Fig. 6). After a 5-day and a 9-day incubation period, *P. koreensis* and *F. pectinovorum* emerged as the dominant bacteria, while *R. kratochvilovae* prevailed as the dominant yeast across all three experimental groups. Notably, there were no significant differences in SynCom overall relative abundance between all three groups (SynCom WT, SynCom mutant, SynCom pseudobactin). To assess whether the presence or absence of pseudobactin had an impact on individual SynCom members, the relative abundance of each strain was separately analyzed, revealing no significant alterations among the different groups (Figs S16 and S17). The results display that, even though it shows strong inhibiting activity on SynCom strains in pairwise interactions, pseudobactin does not affect the SynCom composition or relative abundance of any member *in planta*.

## Discussion

In our study we aimed to investigate microbe–microbe interactions of a synthetic plant leaf community within the epiphytic microbiome compared to pairwise interaction studies. Our findings revealed notable disparities between pairwise interactions observed *in vitro* and those inferred from correlation networks *in planta*. Whereas the correlations in the microbiome were mainly positive, pairwise interactions showed a huge number of inhibitory interactions between SynCom members. The huge repertoire of genes to produce secondary metabolites indicated that pairwise interactions are driven by antimicrobial compounds. Accordingly, we identified pseudobactin from *P. koreensis* as a potent antimicrobial agent against several SynCom members in pairwise interaction experiments. However, pseudobactin had no effect on the SynCom composition *in planta*, mirroring the correlation networks, where *P. koreensis* showed no negative correlation to any SynCom member.

### Pairwise interactions do not affect co-abundance of synthetic community members in the epiphytic microbiome

Pairwise interaction studies via cross-streaking experiments are a common method for the identification of secondary metabolites, especially antimicrobial compounds. AntiSMASH analysis revealed the high potential of *P. koreensis*, *Bacillus altitudinis*, and *Paenibacillus amylolyticus* to produce secondary metabolites, and it was already shown that secondary metabolites produced by plant microbiome members drive strong pairwise interactions [16]. Therefore, it is most likely that the observed inhibitions in the cross-streaking experiments are based on antimicrobials. Why the interactions within our SynCom are not reflected in the correlation networks remains unclear. Even the addition of pure pseudobactin did not alter the SynCom significantly, suggesting that the inhibitory effect of pseudobactin is limited to pairwise interactions and plays a subsidiary role in a microbiome context. Although Getzke *et al*. could show an effect of pyoverdine on the composition of the root microbiome [16], pyoverdines might play a minor role in shaping plant leaf associated communities as compared to root microbiomes. Indeed, while in the rhizosphere iron is limiting and production of siderophores may confer a growth advantage [41, 42] the phyllosphere has higher iron concentrations, decreasing the need for siderophore production [43]. Another possibility might be that pseudobactin was depleted by other SynCom members as this was shown to be the case for lipopeptides produced by *Pseudomonas* spp [44, 45].

We hypothesized that strong inhibitors would have a colonization advantage in communities. Therefore, we anticipated an increase in the relative abundance of the inhibitory strain and a decrease in that of the sensitive strains over the incubation period. However, for *B. altitudinis*, we observed the opposite effect. Although the genome of this strain encodes a high potential to produce secondary metabolites—possibly a reservoir activated only in the presence of certain competitors or pathogens [46, 47]—the strong inhibitor *B. altitudinis* might be constrained by the community. Long-term co-evolution of plant microbiomes has allowed the adjustment of an optimal balance in microbial composition and microbe–microbe interactions [41, 48, 49]. Thus, it is not surprising that a potent member of the SynCom is unable to dominate others, despite its substantial antimicrobial potential. This restraint, for example through suppressed production of antimicrobial compounds, can be further investigated using transcriptomic approaches. Since *B. altitudinis* shows a low relative abundance in the SynCom, the colonization density could have not been sufficient to activate the production of antimicrobials. Tyc *et al*. demonstrated that the antimicrobial activity of certain soil microbiome members is significantly suppressed in co-cultures with commensals compared to monocultures, attributing this to interference with the quorum sensing apparatus or nutrient limitations [50].

Nevertheless, the question remains what *P. koreensis*, *Flavobacterium pectinovorum*, and *Sporobolomyces roseus* have in common to assert their dominant abundance in the SynCom. It is known that both, *S. roseus* and *P. koreensis*, produce exopolysaccharides (EPS), which help them survive harsh environmental conditions [51]. It is furthermore known that EPS are important components of biofilms and that microbiome members can benefit from biofilm producers in their community [52, 53]. *S. roseus* is additionally capable of breaking down leaf surface waxes improving the surface adhesion of organisms on plants [54]. Highlighting their supportive roles, *S. roseus* and *P. koreensis* showed the highest number of positive correlations with the epiphytic microbiome. Their positive linkage strengthens recent findings that adaption by using extracellular metabolites might be a driving force within microbial communities rather than competition by producing antimicrobials [55]. Within the SynCom, *P. koreensis* was again one of the strains counting high number of positive relations but was exceeded by *F. pectinovorum* and *Sphingomonas faeni*. Interestingly, *F. pectinovorum* was also a dominant bacterium in the SynCom *in vitro* and *in planta*, despite being highly sensitive in pairwise interactions. *Flavobacterium* spp. are common members of plant microbiomes and known for their great ability to degrade extracellular macromolecules like starch. Furthermore *Flavobacterium* spp. indirectly promote plant growth, suggesting that they support the microbiome by biotransformation [10, 56, 57]. The high abundance and positive linkage of *F. pectinovorum* in the SynCom strengthens the hypothesis that synergism plays a huge role in shaping microbial communities.

### Correlation networks for the pre-selection of relevant microbe–microbe interactions

Two correlations of SynCom members in the correlation network mirrored the findings in pairwise interactions. *Nocardioides cavernae* and *F. pectinovorum* showed a growth promoting effect in cross-streaking experiments and were positively correlated in the epiphytic microbiome. Moreover, *P. amylolyticus* inhibited *F. pectinovorum in vitro* and the strains were negatively correlated in the microbiome. Whether the pairwise interactions for these strains are truly reflected in correlation networks needs to be further investigated. The mechanistic basis behind the positive connections of *N. cavernae* and *F. pectinovorum* in the plant microbiome is not yet understood. For *P. amylolyticus* strains it was already shown that they are able to produce polymyxin antibiotics [58, 59]. Interestingly, the *P. amylolyticus* strain from the SynCom carries a gene cluster with 100% similarity to polymyxin B. The production of the compound might explain the antimicrobial activity in pairwise interactions since it is potent against gram negative bacteria like *F. pectinovorum* [60]. Whether the inhibitory interaction is so dominant to be observed within the epiphytic microbiome in correlation networks remains unknown, but it is a promising start for future investigations. Furthermore, it shows that correlation networks are promising methods for preselecting microbe–microbe interactions involved in microbiome shaping. Several publications successfully used bottom–up methods such as pairwise interaction analysis for further investigations in microbial communities and microbiomes [16, 17, 61]. As shown by Sun *et al*., pairwise interaction studies were successfully combined with genome scale metabolic modeling to explain positive and negative correlations in a synthetic biofilm community [62].

In correlation networks, OTUs for four SynCom members, including *B. altitudinis*, were absent due to the read count threshold applied in the study. We hypothesize that these strains may have been outcompeted in their niches by closely related species, as previously observed for the gut microbiome [63]. The identification of the SynCom members as part of the core microbiome in the original study, from which the SynCom was assembled [13], and the low abundance of *B. altitudinis* and others in the correlation network raw data [24], underscore the spatiotemporal dependency of microbiome compositions. Therefore, it is essential to recognize that correlation networks reflect not only microorganismic interactions but also environmental influences. Mahmoudi *et al*. demonstrated that up to 25% of correlations in the *A. thaliana* leaf microbiome can be attributed to environmental factors. However, the majority of correlations were not explainable by environmental factors investigated by the authors suggesting underlying microbe–host and microbe–microbe interactions [24].

Our findings indicate that when investigating microbiome interactions on a pairwise basis, there is a high likelihood that these interactions prove to be less significant than expected. As soon as three and more interaction partners exist together in a model system, the complexity of the interaction network increases drastically, limiting the meaningfulness of pairwise interaction approaches [64]. Therefore, beyond pairwise interaction methods especially computational approaches are getting more and more into the focus of research [65, 66]. Our results demonstrate the limitations of pairwise interaction approaches and suggest the use of microbiome-wide studies like correlation networks for the investigation of dynamics shaping and stabilizing microbial communities. Furthermore, the use of synthetic communities can give insights into the importance of a compound or strain in a microbiome context and therefore is a promising method for investigating microbiome dynamics.

## Supplementary Material

supplemental_information_revised_ycae117

## Data Availability

The raw datasets generated from amplicon sequencing for the relative abundance of SynCom members *in vitro* and *in planta* are available in the Zenodo repository (Strong pairwise interactions do not drive interactions in a plant leaf associated microbial community), [https://zenodo.org/records/12795858] DOI: 10.5281/zenodo.12795858. The datasets for the visualization of the correlation networks are available in the Zenodo repository (strong pairwise interactions do not drive interactions in a plant leaf associated microbial community), [https://zenodo.org/records/12795858], DOI: 10.5281/zenodo.12795858. The OTU data and workflows used for correlation network calculation are available in the Zenodo repository (strong pairwise interactions do not drive interactions in a plant leaf associated microbial community), [https://zenodo.org/records/12795858], DOI: 10.5281/zenodo.12795858. All raw data for correlation networks based on co-abundance analyzed during this study are included in the published article of Mahmoudi *et al*. and its supplementary information files.
