# Supplementary material for: Strong pairwise interactions do not drive interactions in a plant leaf associated microbial community
Source: ISME Commun. 2024 Oct 4;5(1):ycae117. doi: 10.1093/ismeco/ycae117 (PMC12551452; doi:10.1093/ismeco/ycae117)
Supplement: supplemental_information_revised_ycae117 [file supplemental_information_revised_ycae117.docx]

Supplemental information to the article

Strong Pairwise Interactions do not Drive Interactions in a Plant Leaf Associated Microbial Community

Franziska Höhn^1,3^, Dr. Vasvi Chaudhry^2^, Dr. Caner Bagci^1^, Maryam Mahmoudi^2^, Elke Klenk^2^, Lara Berg^1,3^, Dr. Paolo Stincone^2,3^, Dr. Chambers C. Hughes^3,4,6^, Dr. Daniel Petras^3,5^, Prof. Heike Brötz-Oesterhelt^3,4,6^, Prof. Eric Kemen^2,3*^, Prof. Nadine Ziemert^1,3,4*^

^1^Translational Genome Mining for Natural Products, Interfaculty Institute of Microbiology and Infection Medicine (IMIT) and Institute for Bioinformatics and Medical Informatics (IBMI), University of Tübingen, Tübingen, Germany

^2^Center for Plant Molecular Biology (ZMBP), Interfaculty Institute of Microbiology and Infection Medicine (IMIT), University of Tübingen, Tübingen, Germany

^3^ Cluster of Excellence Controlling Microbes to Fight Infections (CMFI), University of Tübingen, Tübingen, Germany

^4^German Centre for Infection Research (DZIF), Partner Site Tübingen, Tübingen, Germany

^5^Department of Biochemistry, University of California Riverside, Riverside, USA

^6^Department of Microbial Bioactive Compounds, Interfaculty Institute of Microbiology and Infection Medicine (IMIT), University of Tübingen, Tübingen, Germany

**List of figures**

[Figure S1: Pictures of cross-streaking experiments as examples. 5](#_Toc172809035)

[Figure S2: Growth curves of P. koreensis WT and pseudobactin mutant in MM9 medium 6](#_Toc172809036)

[Figure S3: Total number of OTUs connected to SynCom members by edges in correlation networks based on co-abundance. 7](#_Toc172809037)

[Figure S4: Relative abundance of B. altitudinis after 0 days and 5 days incubation. 10](#_Toc172809038)

[Figure S5: Time-fold increase or decrease of relative abundance of SynCom members over incubation time 10](#_Toc172809039)

[Figure S6: Structure of pseudobactin A TFA salt (1) with 1H and 13C chemical shift assignments in D2O at 700 MHz 11](#_Toc172809040)

[Figure S7: ^1^H NMR (D2O, 700 MHz) of pseudobactin A TFA salt (1) 12](#_Toc172809041)

[Figure S8: ^13^C NMR (D2O, 175 MHz) of pseudobactin A TFA salt (1) 13](#_Toc172809042)

[Figure S9: COSY NMR (D2O, 700 MHz) of pseudobactin A TFA salt (1) 14](#_Toc172809043)

[Figure S10: TOCSY NMR (D2O, 700 MHz) of pseudobactin A TFA salt (1) 15](#_Toc172809044)

[Figure S11: Figure S5 HSQC NMR (D2O, 700 MHz) of pseudobactin A TFA salt (1) 16](#_Toc172809045)

[Figure S12:HMBC NMR (D2O, 700 MHz) of pseudobactin A TFA salt (1) 17](#_Toc172809046)

[Figure S13: HR¬MS spectrum of pseudobactin A TFA salt (1) 18](#_Toc172809047)

[Figure S14: Pseudobactin production and activity in P. koreensis WT and ΔpvdI/J mutant. 19](#_Toc172809048)

[Figure S15: Growth curves of SynCom members in presence or absence of pseudobactin. 20](#_Toc172809049)

[Figure S16: T-test of each SynCom member for the experiment (Fig.6) after 5 days of incubation: 21](#_Toc172809050)

[Figure 17: T-test of each SynCom member for the experiment (Fig.6) after 9 days of incubation: 21](#_Toc172809051)

**Table Index:**

Table S1: MM9 medium used in growth curves for investigating the effect of pseudobactin on SynCom members. 3

Table S2: Enriched MM9 medium used in growth curves for investigating the effect of pseudobactin on SynCom members. 3

Table S3: MM9/7 defined minimal agar for 16S rRNA/ITS2 amplicon sequencing of SynCom strains in vitro.. 4

Table S4: List of Primers used for the creation of the pseudobactin deletion mutant ΔpvdI/J. 5

Table S5: OTUs representing SynCom members which were removed from correlation network. 6

Table S6: AntiSMASH biosynthetic gene cluster prediction of SynCom strains and similarity to known BGCs. 8

**Table S1: MM9 medium used in growth curves for investigating the effect of pseudobactin on SynCom members**. P. koreensis WT and pseudobactin mutant were grown in MM9 to obtain WT and ∆pvdI/J supernatant. The medium was highly iron-limited and used for growth curves of A. humicola and S. faeni.

| Medium additive | Standard MM9 | WT + MM9 | ∆pvdI/J + MM9 | WT + FeSO4 + MM9 | *∆pvdI/J* + Pseudobactin + MM9 |
| --- | --- | --- | --- | --- | --- |
| MM9 medium | 100 ml | 90 ml | 90 ml | 90 ml | 90 ml |
| WT supernatant | - | 10 ml | - | 10 ml |  |
| *∆pvdI/J* supernatant | - |  | 10 ml | - | 10 ml |
| FeSO_4_ (1.5 mg/ml) | - |  |  | 100 µl |  |
| Pure Pseudobactin (100 µg/ml) | - |  |  |  | 100 µl |

**Table S2: Enriched MM9 medium used in growth curves for investigating the effect of pseudobactin on SynCom members**. P. koreensis WT and pseudobactin mutant were grown in MM9 to obtain WT and ∆pvdI/J supernatant. Strains not able to grow in iron-limited MM9 medium were grown in enriched MM9 containing low amounts of optimal growth medium NB (for bacteria) or PDB (for yeasts). Enriched MM9 medium was used for growth curves of B. altitudinis, A. fastidiosum, P. amylolyticus, M. proteolyticum, S. roseus, R. kratchovilovae.

| Medium additive | Standard enriched MM9 | WT + MM9 | ∆pvdI/J + MM9 | WT + FeSO4 + MM9 | *∆pvdI/J* + pseudobactin + MM9 |
| --- | --- | --- | --- | --- | --- |
| MM9 medium | 80 ml | 70 ml | 70 ml | 70 ml | 70 ml |
| NB/PDA | 20 ml | 20 ml | 20 ml | 20 ml | 20 ml |
| WT supernatant | - | 10 ml | - | 10 ml |  |
| *∆pvdI/J* supernatant | - |  | 10 ml | - | 10 ml |
| FeSO_4_ (1.5 mg/ml) | - |  |  | 100 µl |  |
| Pure pseudobactin (100 µg/ml) | - |  |  |  | 100 µl |

**Table S3: MM9/7 defined minimal agar for 16S rRNA/ITS2 amplicon sequencing of SynCom strains in vitro**. MM9 medium was modified as shown in the table to obtain a defined minimal agar suitable for amplicon sequencing and inspired by the plant leaf surface.

| Solution | Chemical | Volume |
| --- | --- | --- |
| Pre autoclave solution in 950 ml | KH_2_PO_4_ | 0.30 g |
|  | NaCl | 0.50 g |
|  | NH_4_Cl | 1.00 g |
|  | Agar | 15.00 g |
| Autoclave at 121 °C, 20 min | | |
| Post autoclave solution (filter sterilize 0.2 µm and add to pre autoclave solution) | Glucose 20 % | 10.00 ml |
|  | MgSO_4_ 1 M | 1.00 ml |
|  | CaCl_2_ 100 mM | 1.00 ml |
|  | amino acid solution | 30.00 ml |
|  | Trace element solution | 10.00 ml |
| Amino acid solution preparation filter sterilized (0.2µm)   - Mix equal volumes of each solution I-VI | | |
| Solution I (in 100 ml dH_2_O) | Phe | 0.99 g |
|  | Lys | 1.10 g |
|  | Arg | 2.50 g |
| Solution II (in 100 ml dH_2_O) | Gly | 0.20 g |
|  | Val | 0.70 g |
|  | Ala | 0.84 g |
|  | Trp | 0.41 g, |
| Solution III (in 100 ml dH_2_O) | Thr | 0.71g |
|  | Ser | 8.40 g |
|  | Pro | 4.60 g |
|  | Asn | 0.96 g |
| Solution IV (in 90 ml dH_2_O + 10 ml HCl (36 %)) | Asp (free acid) | 1.04 g |
|  | Gln | 14.60 g |
| Solution V (dissolve K.Glu in 80 ml dH_2_O, add rest and fill up to 100 ml with dH_2_O) | K.Glu | 18.70 g |
|  | Tyr | 0.36 g |
|  | NaOH | 4.00 g |
| Solution VI (in 100 ml dH_2_O) | Ile | 0.79 g |
|  | Leu | 0.77 g |
| Trace element solution preparation (filter sterilized 0.2 µm) | | |
| EDTA-solution (in 800 ml dH_2_O, pH 7.5) | EDTA | 5.00 g |
| Final solution (fill up to 1 L with dH_2_O) | FeCl_3_ - 6 H_2_O | 0.83 g |
|  | ZnCl_2_ | 84.00 mg |
|  | CuCl_2_ - 2H_2_O | 13.00 mg |
|  | CoCl_2_ - 2H_2_O | 10.00 mg |
|  | H_3_BO_3_ | 10.00 mg |
|  | MnCl_2_ - 4H_2_O | 1.60 mg |


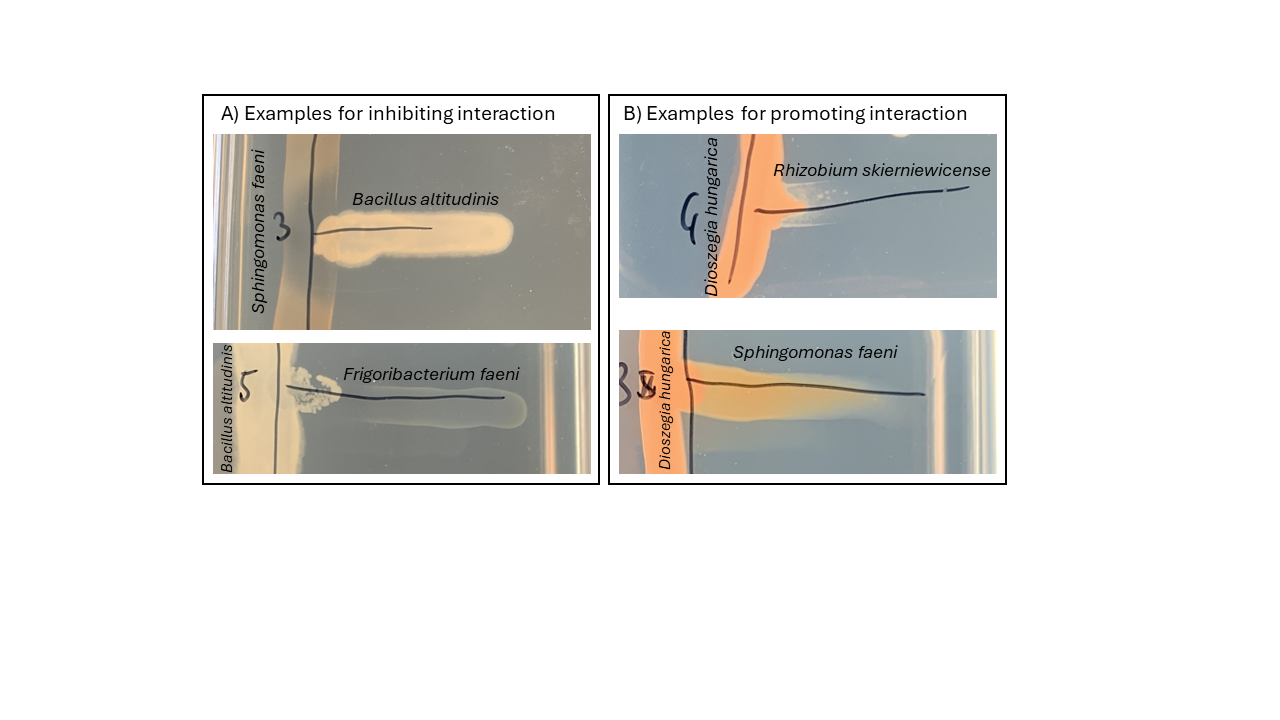


**Figure S1: Pictures of cross-streaking experiments as examples.** A) Inhibiting interactions between SynCom members were visually observed by the presence of inhibition zones. The here shown cross-streakings were performed on NA. B) Promotive interactions were visually observed by better growth in the contact zone. The here shown cross-streakings were performed on PDA.

**Table S4: List of Primers used for the creation of the pseudobactin deletion mutant ΔpvdI/J.** The deletion was performed as described by Huang et al. * Primers can additionally be used for the verification of a successful deletion.

| Primer | Sequence 5´-> 3´ | purpose |
| --- | --- | --- |
| pEX18seq_fw | GGATGTGCTGCAAGGCGATTAAG | Forward primer for verification of single cross-over **upstream of *pvdI.*** |
| pEX18seq_rv | GGCTCGTATGTTGTGTGGAATTGTG | Reverse primer for verification of single cross-over **downstream of *pvdJ.*** |
| pvd_upstream_f | GATCCCCGGGTACCGAGCTCGATG  AATGCCGCAGACGCACAGAAAC | Forward primer for the amplification of the left part of the deleted gene region for integration into vector. Region is **upstream from *pvdI****. ** |
| pvd_upstream_rv | CAGCATCGCCAATGCCGCTGGCCGT  TCGCTGTCGGCGAGCATC | Reverse primer for the amplification of the left part of the deleted gene region for integration into vector. Region is **upstream from *pvdI****.* |
| pvd_downstream_f | GATGCTCGCCGACAGCGAACGGCCA  GCGGCATTGGCGATGCTGCC | Forward primer for the amplification of the right part of the deleted gene region for integration into vector. Region is **downstream from** *pvdJ.* |
| pvd_downstream_rv | GAAACAGCTATGACCATGATTACGCG  AGGGATAGACGTTTGAGGGCCTCCAGTG | Reverse primer for the amplification of the right part of the deleted gene region for integration into vector. Region is **downstream from *pvdJ****.* |
| pvdI+pvdJ_before | CGTTCGAAGGGCCGCGCAAAGTC | Forward primer for the verification of deletion success. |
| pvdI+pvdJ_after | GCGATGCTGCGTTCCAGTGCCG | Reverse primer for the verification of deletion success. |
| behind_up_rw | GAACAGGCTTTGCACGCTGGTAAAC | Reverse primer for verification of single cross-**over upstream of *pvdI. **** |
| before_down_f | GAACGCTTGCTGCACATGCTC | Forward primer for verification of single cross-over **downstream of *pvdJ.*** |

**Figure S2: Growth curves of P. koreensis WT and pseudobactin mutant in MM9 medium** Similar growth for P. koreensis WT and ΔpvdI/J mutant was observed in MM9 medium. This was important, since the supernatant was used in pseudobactin interaction studies.

**Table S5: OTUs representing SynCom members which were removed from correlation network**. The OTUs showed highest BlastN similarity to the 16S rRNA/ITS2 sequence of the named SynCom member. OTUs showing < 10 reads per sample and/or occurrence in < 5 sample

| **OTU** | **related SynCom strain** | **BlastN similarity** | **samples with OTU occurence** | **samples with > 10 reads** |
| --- | --- | --- | --- | --- |
| Otu002983 | *B. altitudinis* | 98.70% | 9 | 3 |
| Otu004835 | *F. faeni* | 100.00% | 35 | 0 |
| Otu02956 | *D. hungarica* | 100.00% | 29 | 0 |
| Otu00955 | *R. kratchovilovae* | 100.00% | 3 | 2 |

**Figure S3: Total number of OTUs connected to SynCom members by edges in correlation networks based on co-abundance**. Total positive correlations (cor > 0) (light blue) and negative correlations (cor < 0) (red) of SynCom members to the epiphytic microbiome of A. thaliana. Positive (dark blue) and negative (organe) correlations of SynCom members to each other extracted of the whole correlation network. For P. amylolyticus edge numbers of OTU001595 were used.

**Table S6: AntiSMASH biosynthetic gene cluster prediction of SynCom strains and similarity to known BGCs**. AntiSMASH 7.0 was used for the identification of BGCs of SynCom members and their similarity to known clusters. *Two split NRPS BGCs of P. koreensis

| **Strain** | **Cluster prediction** | **Most similar known cluster** | **similarity** |
| --- | --- | --- | --- |
| 1. *fastidiosum* | redox-cofactor |  |  |
|  | NI-siderophore | desferrioxamine E | 75 % |
|  | RiPP-like |  |  |
|  | NAPAA | e-Poly-L-Lysine | 100 % |
| 1. *humicola* | NRPS-like | SLI- 2138 | 11 % |
|  | type 3 PKS | pentalenolactone | 15 % |
|  | betalactone | microansamycin | 7 % |
|  | NAPAA | stenothricin | 31 % |
|  | NAPAA |  |  |
|  | RRE-containing |  |  |
| 1. *altitudinis* | betalactone | - | - |
|  | RiPP-like |  |  |
|  | type 3 PKS |  |  |
|  | NRPS | lichenysin | 85 % |
|  | NRP-metallophore | bacillibactin | 80 % |
|  | RiPP-like |  |  |
|  | type 1 PKS / NRPS | zwittermycin A | 18 % |
|  | betalactone | fengycine | 53 % |
|  | terpene |  |  |
|  | NRPS-like | locillomycin | 21 % |
|  | NI-siderophore | schizokinen | 60 % |
|  | RRE-containing |  |  |
| *F. pectinovorum* | arylpolyene/resorcinol | flexirubin | 91 % |
|  | terpene | carotenoid | 28 % |
|  | betalactone |  |  |
| *F. faeni* | type 3 PKS | funisamine | 7 % |
|  | NI-siderophore | FW0622 | 37 % |
|  | terpene | carotenoid | 50 % |
| *M. aurea* | terpene |  |  |
|  | RiPP-like | paulomycin | 3 % |
|  | arylpolyene | APE Vf | 35 % |
|  | hserlactone |  |  |
|  | hserlactone, RRe-containing |  |  |
|  | hydrogen-cyanide |  |  |
|  | terpene | carotenoid | 100 % |
| *M. goesingense* | terpene | carotenoid | 100 % |
|  | RiPP-like |  |  |
|  | redox-cofactor |  |  |
|  | type 1 PKS | oryzanaphthopyran A | 6 % |
|  | NRP-metallophore | taiwachelin | 22 % |
|  | hserlactone |  |  |
|  | terpene |  |  |
|  | terpene |  |  |
|  | type 1 PKS/ NRPS |  |  |
|  | NAPAA |  |  |
| *M. proteolyticum* | betalactone | microansamycin | 7 % |
|  | terpene | carotenoid | 21 % |
|  | NAPAA | e-Poly-L-Lysin | 100 % |
|  | type 3 PKS |  |  |
| *N. cavernae* | terpene | carotenoid | 14 % |
|  | Betalactone / NRPS-like | formicamycins A-M | 4 % |
|  | type 3 PKS | alkylresorcinol | 100 % |
| *P. amylolyticus* | type 3 PKS |  |  |
|  | type 3 PKS | corynecin II | 13 % |
|  | NRPS-like |  |  |
|  | lassopeptide | paeninodin | 60 % |
|  | proteusin |  |  |
|  | NI-siderophore |  |  |
|  | trans-AT PKS / NRPS | pellasoren | 33 % |
|  | trans-AT PKS / NRPS | paenilipoheptin | 23 % |
|  | terpene | carotenoid | 33 % |
|  | Opine-like-metallophore | bacillopaline | 100 % |
|  | lanthipeptide-class-ii | Gramicidin S | 15 % |
|  | lanthipeptide-class-iv |  |  |
|  | NRPS | polymyxin | 100 % |
| *P. koreensis* | NAGGN |  |  |
|  | NRPS | Pf-5 pyoverdine* | 21 % |
|  | arylpolyene | APE Vf | 40 % |
|  | NRPS-like | fragin | 37 % |
|  | RiPP-like |  |  |
|  | NRP-metallophore | Pf-5 pyoverdine* | 10 % |
|  | RiPP-like |  |  |
|  | RiPP-like |  |  |
|  | betalactone | fengycin | 13 % |
|  | hydrogen-cyanide | hydrogen cyanide | 100 % |
|  | redox-cofactor | lankacidin C | 13 % |
|  | RiPP-like |  |  |
| *R. skierniewicense* | terpene |  |  |
|  | arylpolyene | persiamycin A | 5 % |
|  | lanthipeptide-class V |  |  |
|  | NI-siderophore | desferrioxamine E | 50 % |
|  | betalactone | xantholipin | 4 % |
|  | NI-siderophore | roseobactin | 50 % |
|  | thioamitides |  |  |
|  | betalactone |  |  |
|  | hserlactone |  |  |
|  | hydrogen-cyanide |  |  |
|  | hserlactone |  |  |
|  | type 1 PKS |  |  |
| *S. faeni* | RiPP-like |  |  |
|  | terpene | carotenoid | 50 % |
|  | type 3 PKS |  |  |
|  | redox-cofactor | lankacidin C | 13 % |
| 1. *hungarica* | terpene |  |  |
|  | NRPS-like |  |  |
|  | NRPS-like |  |  |
|  | terpene |  |  |
|  | NRPS-like |  |  |
|  | terpene |  |  |
| *R. kratchovilovae* | NRPS-like |  |  |
|  | NRPS |  |  |
|  | terpene |  |  |
|  | betalactone |  |  |
|  | terpene |  |  |
| *S. roseus* | NRPS-like |  |  |
|  | terpene |  |  |
|  | betalactone |  |  |
|  | NRPS |  |  |


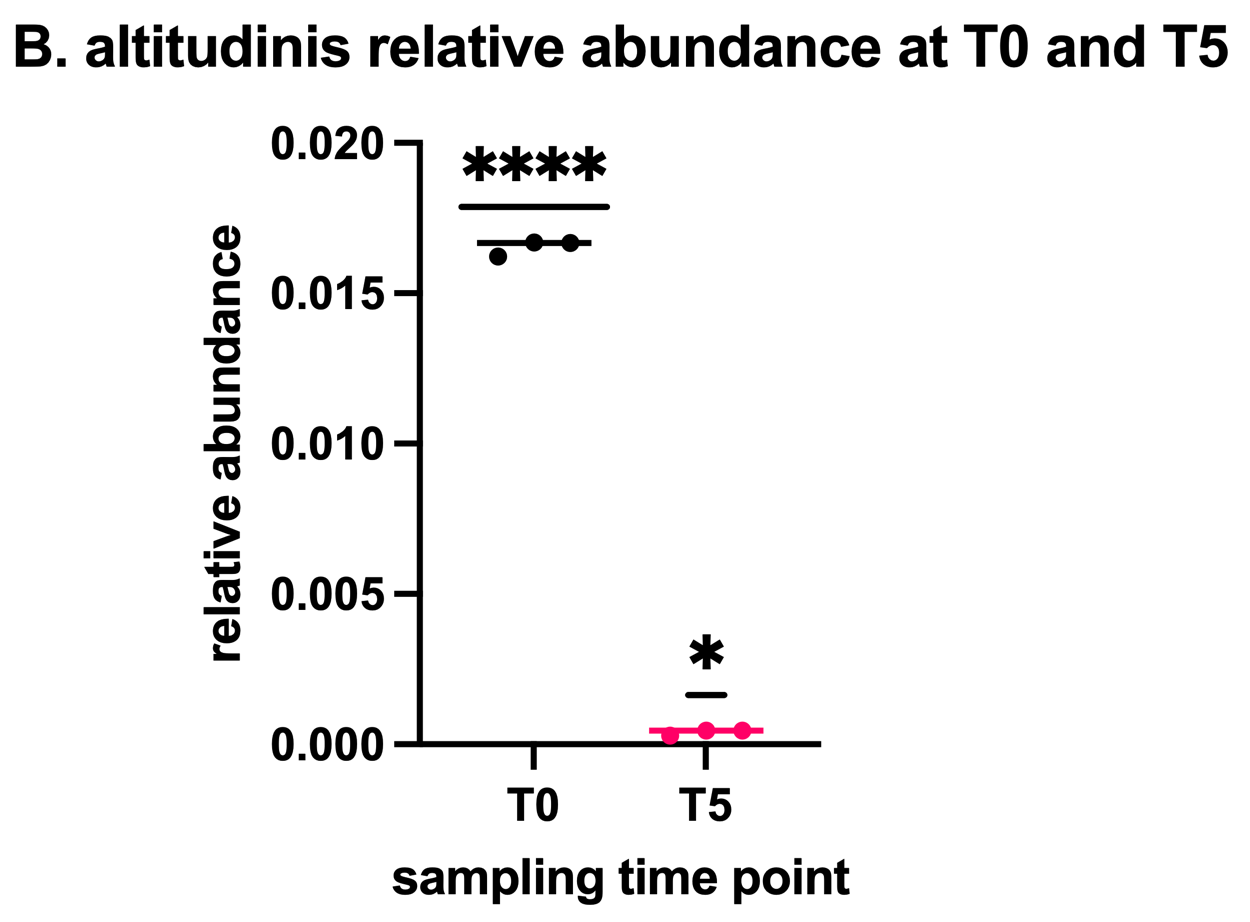


**Figure S4: Relative abundance of B. altitudinis after 0 days and 5 days incubation.** The decrease of relative abundance of B. altitudinis, when grown in the SynCom on mm9/7 agar at inoculation and after 5 days of incubation at 22 °C.


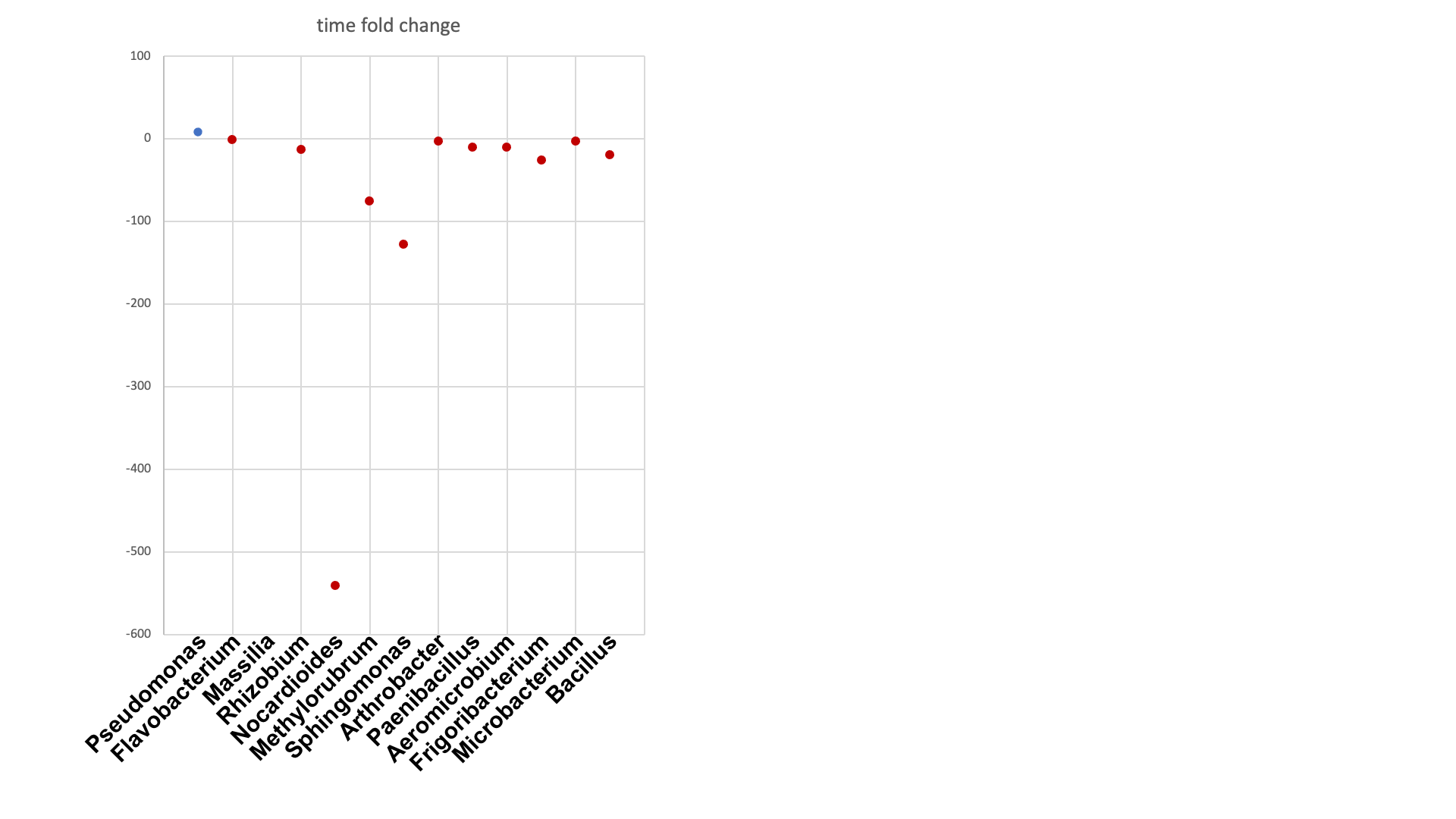


**Figure S5: Time-fold increase or decrease of relative abundance of SynCom members over incubation time** The calculation of the increase (blue) or decrease (red) was based on the relative abundance after 0 and 5 days of incubation in a whole SynCom co-culture (0.2 OD600 of each strain). The SynCom was cultured on MM9/7 agar at 22 °C and relative abundance was investigated by 16S rRNA/ITS2 MiSeq illumina amplicon sequencing.


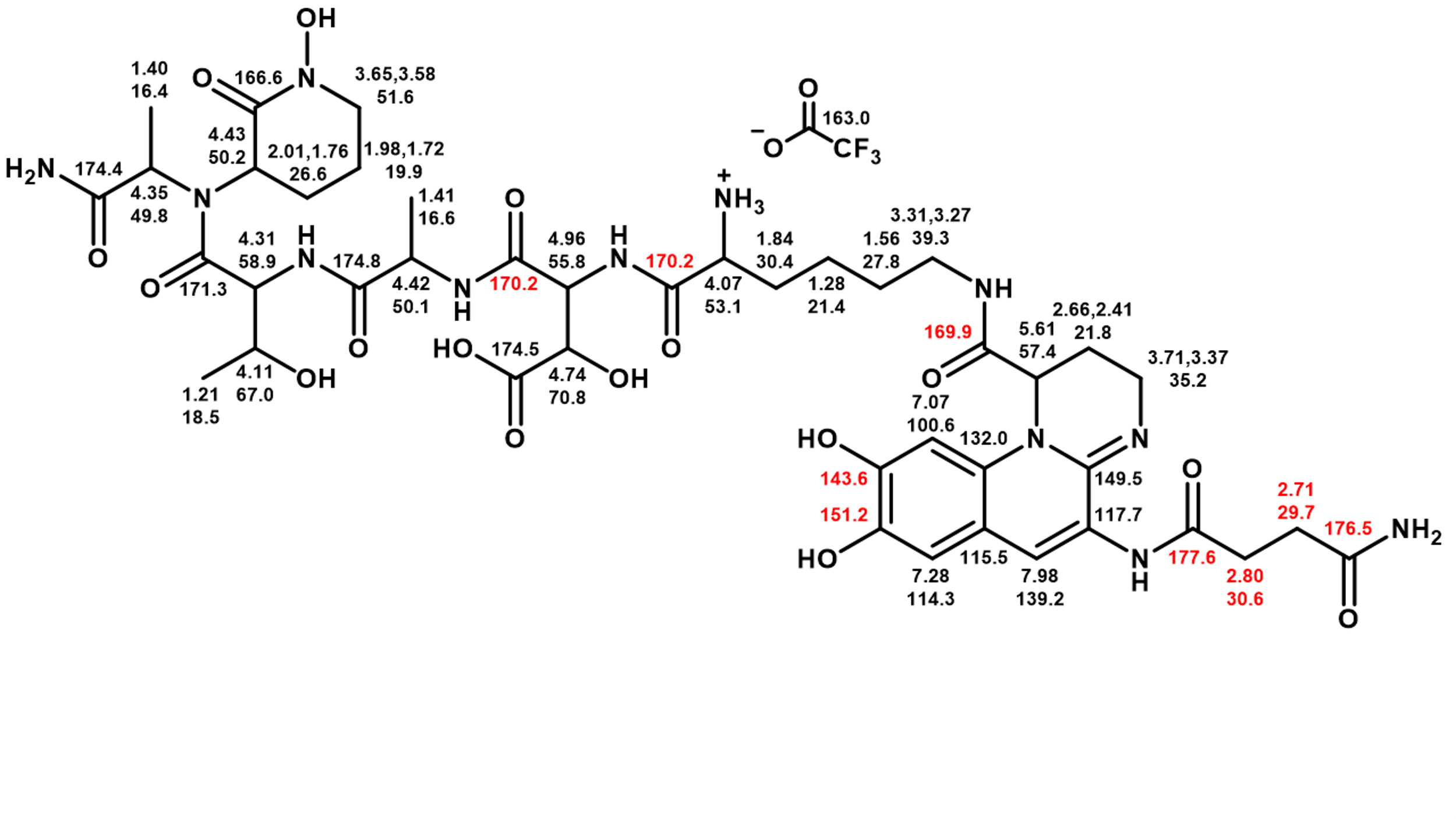


**Figure S6: Structure of pseudobactin A TFA salt (1) with 1H and 13C chemical shift assignments in D2O at 700** **MHz** NB: 1) ^1^H NMR chemical shifts are in good agreement with Teintze, M.; Leong, J. Structure of pseudobactin A, a second siderophore from plant growth promoting Pseudomonas B10. Biochemistry 1981, 20, 6457–6462. DOI:10.1021/bi00525a026. 2) Some uncertainty is associated with the assignments in red.


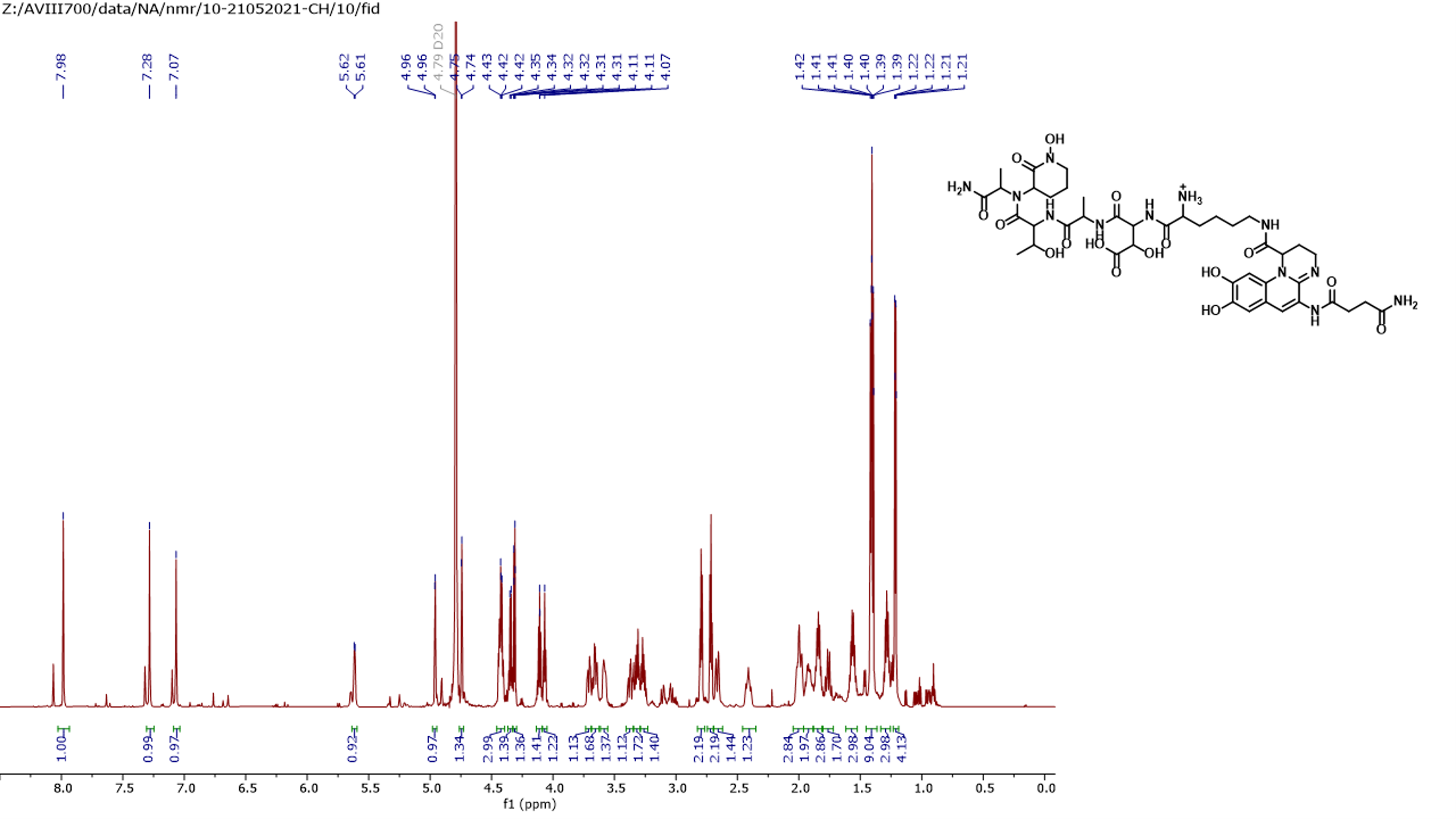


**Figure S7: ^1^H NMR (D2O, 700 MHz) of pseudobactin A TFA salt (1)**


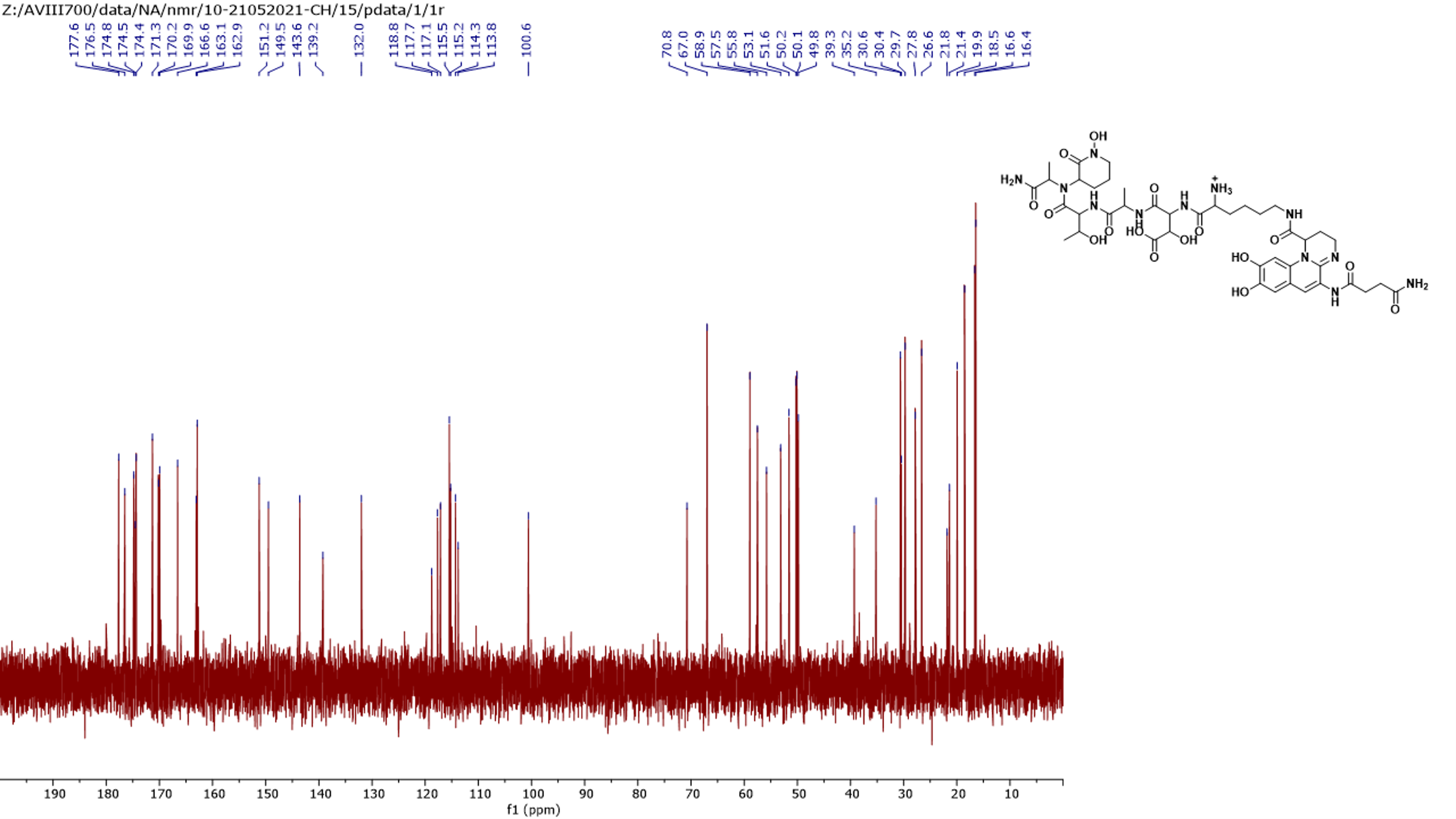


**Figure S8: ^13^C NMR (D2O, 175 MHz) of pseudobactin A TFA salt (1)**


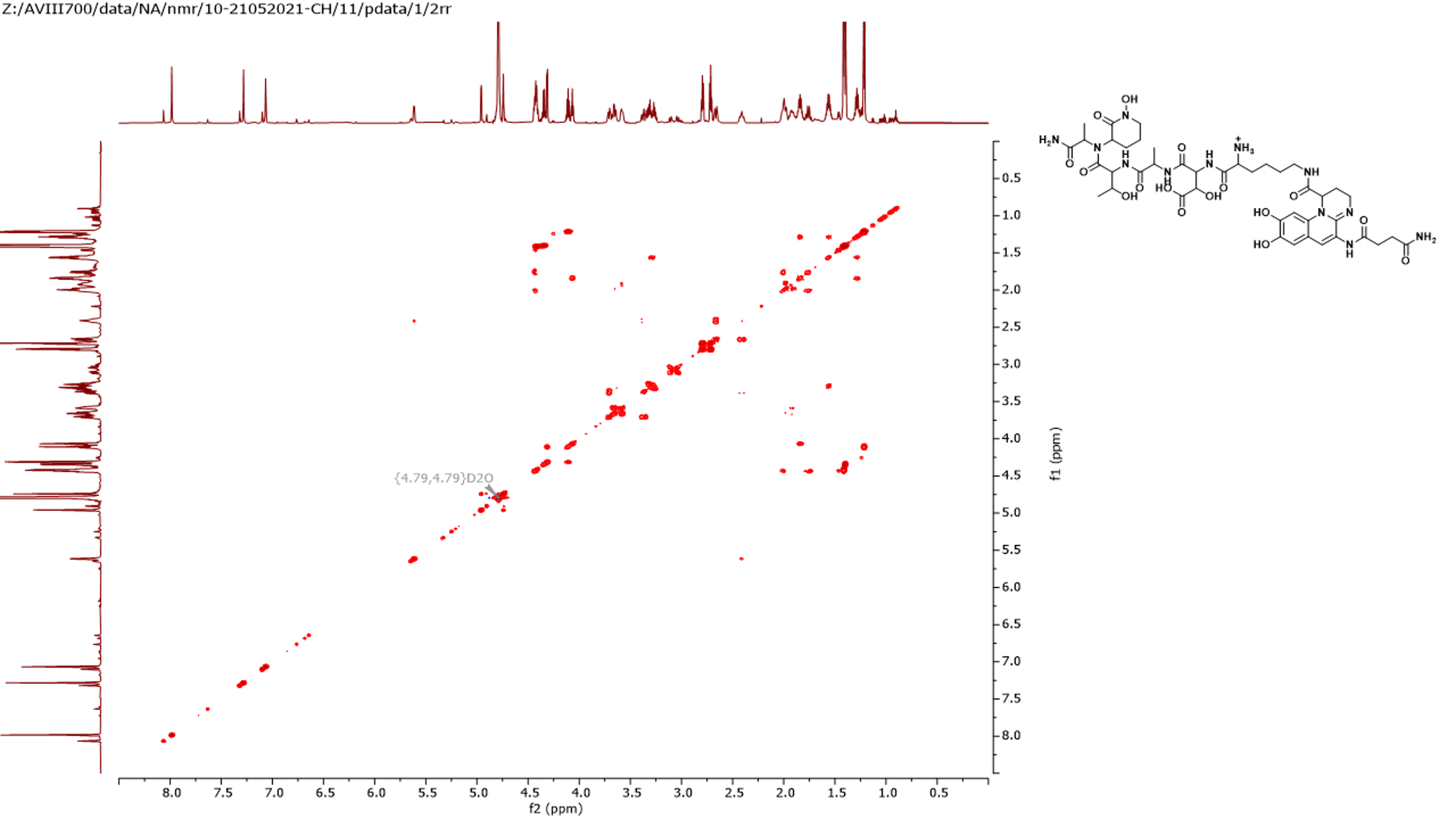


**Figure S9: COSY NMR (D2O, 700 MHz) of pseudobactin A TFA salt (1)**


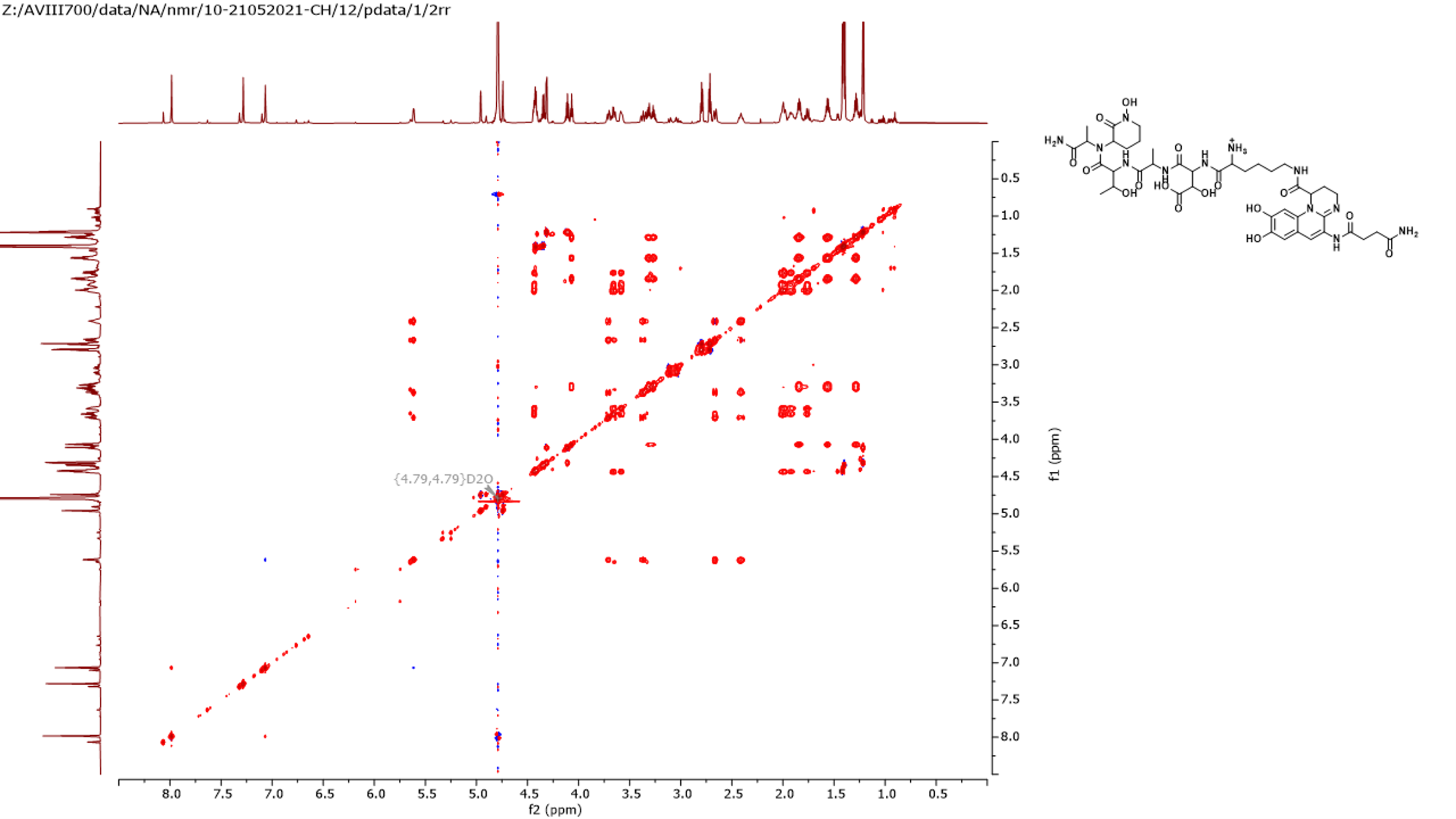


**Figure S10: TOCSY NMR (D2O, 700 MHz) of pseudobactin A TFA salt (1)**


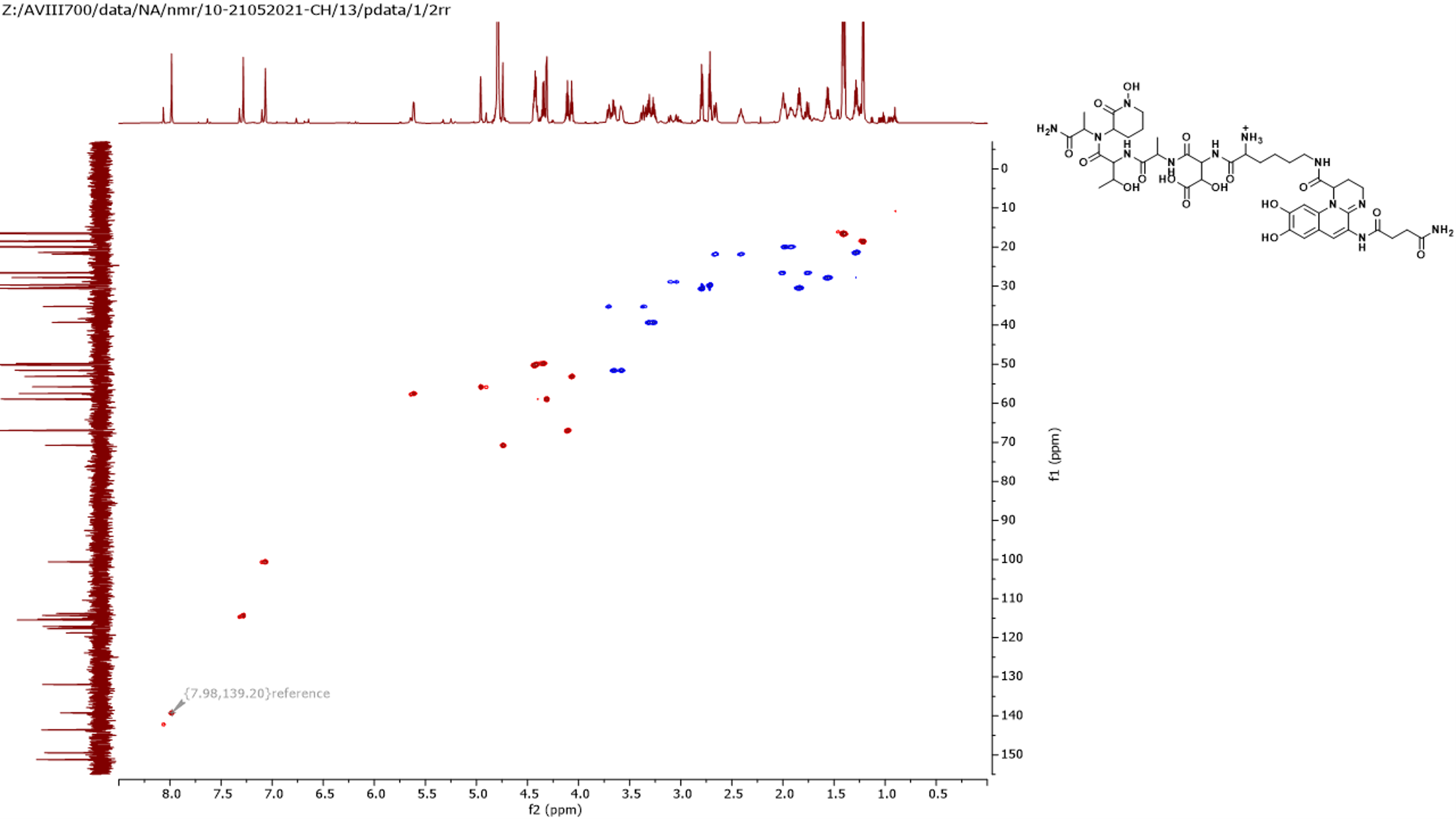


**Figure S11: HSQC NMR (D2O, 700 MHz) of pseudobactin A TFA salt (1)**


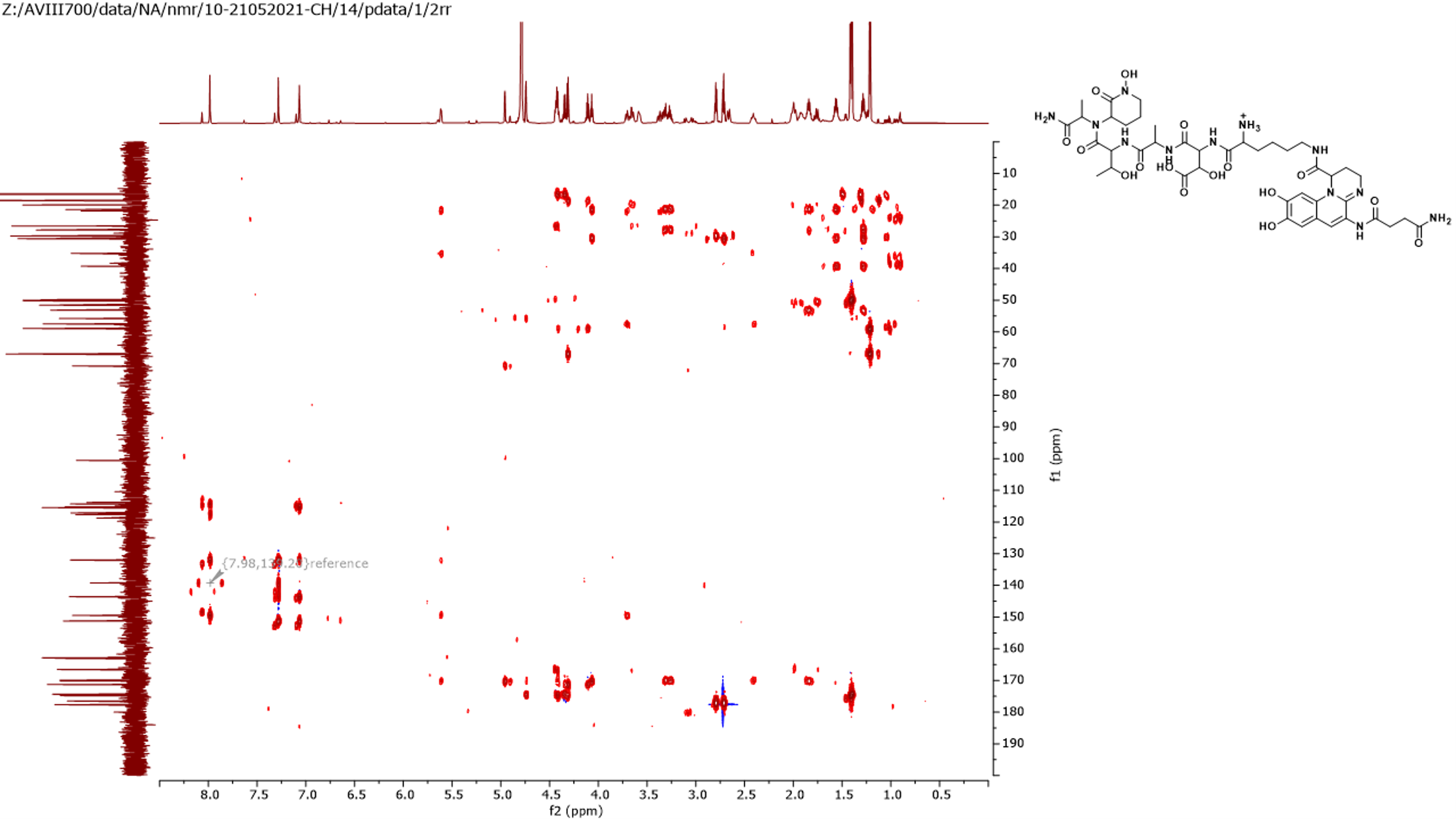


**Figure S12:HMBC NMR (D2O, 700 MHz) of pseudobactin A TFA salt (1)**


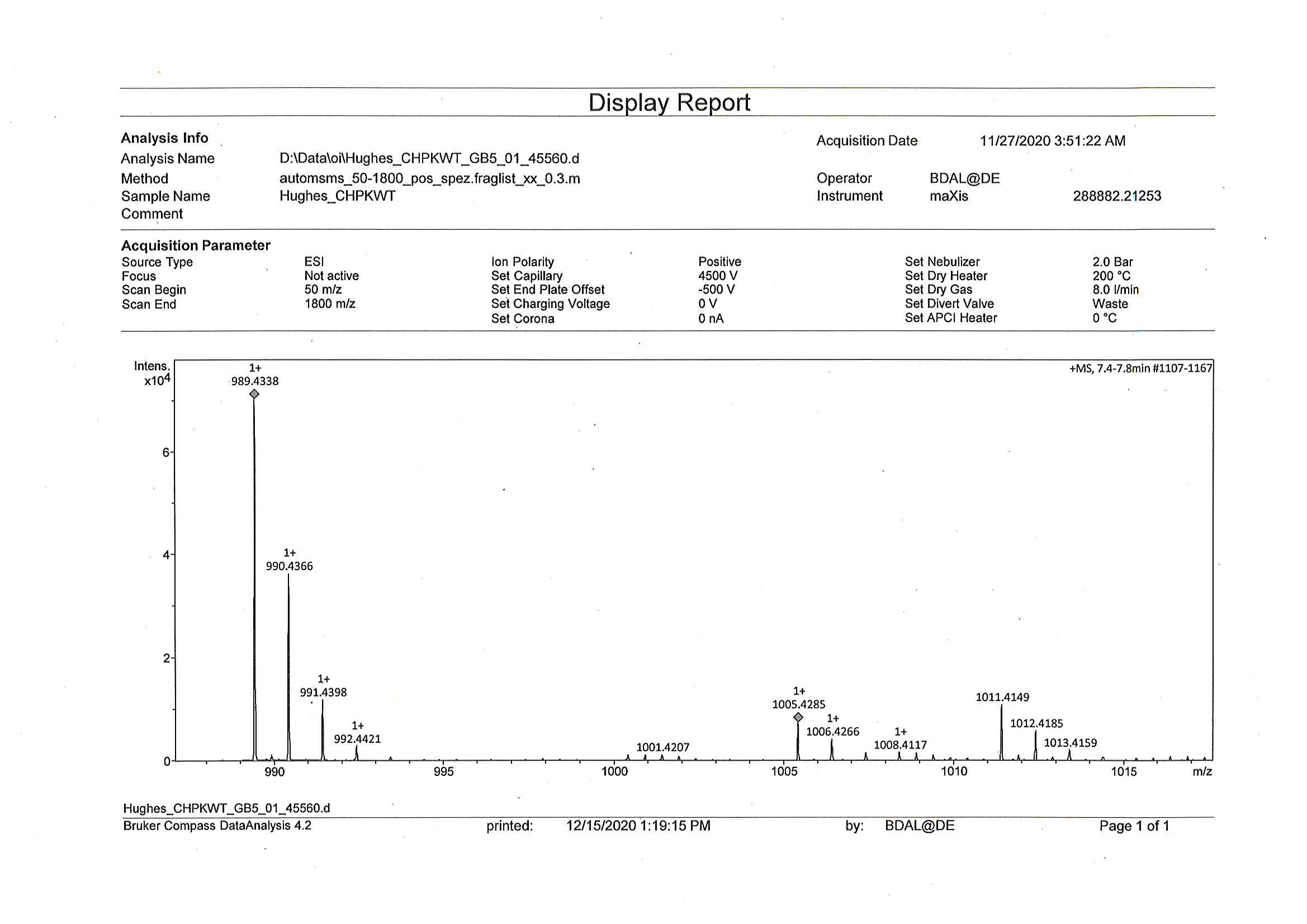


*m/z* [M+H]^+^ = 989.4338, calcd for C_42_H_61_N_12_O_16_, 989.4323

**Figure S13: HR¬MS spectrum of pseudobactin A TFA salt (1)**


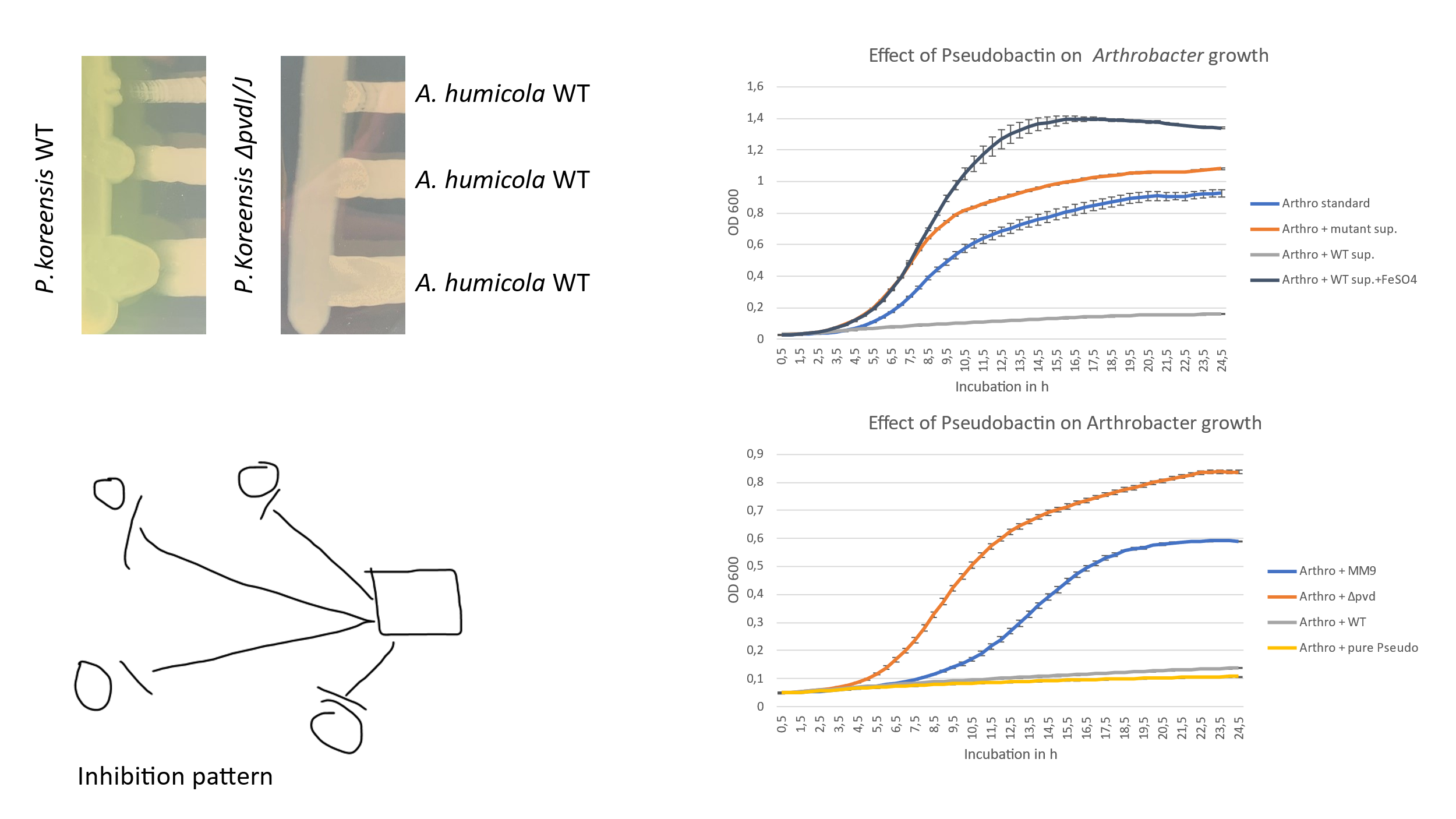


1. **Cross-streaking experiment of *P. koreensis* WT and mutant**
2. **HPLC-MS measurement of *P. koreensis* WT and mutant supernatant**

*P. koreensis* WT

*ΔpvdI/J* mutant

Pseudobactin [M+H]^+^


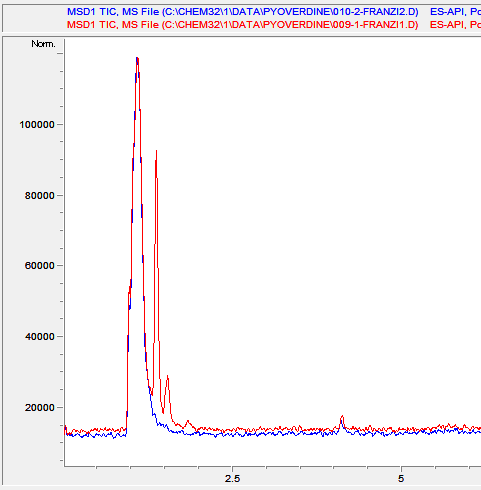


**Figure S14: Pseudobactin production and activity in P. koreensis WT and ΔpvdI/J mutant**. a) HPLC-MS of P. koreensis WT supernatant (red) and ΔpvdI/J supernatant (blue) was prepared as explained in material and methods section. A clear peak for the mass of pseudobactin is visible in WT supernatant and no peak can be seen in mutant supernatant. b) Cross-streaking experiments on f-base agar for the detection of pseudobactins´ inhibitory interaction with A. humicola. Inhibition zones and fluorescence of pseudobactin can be seen on the left (P. koreensis WT). Loss of inhibitory activity and fluorescence is observed for the mutant (right).


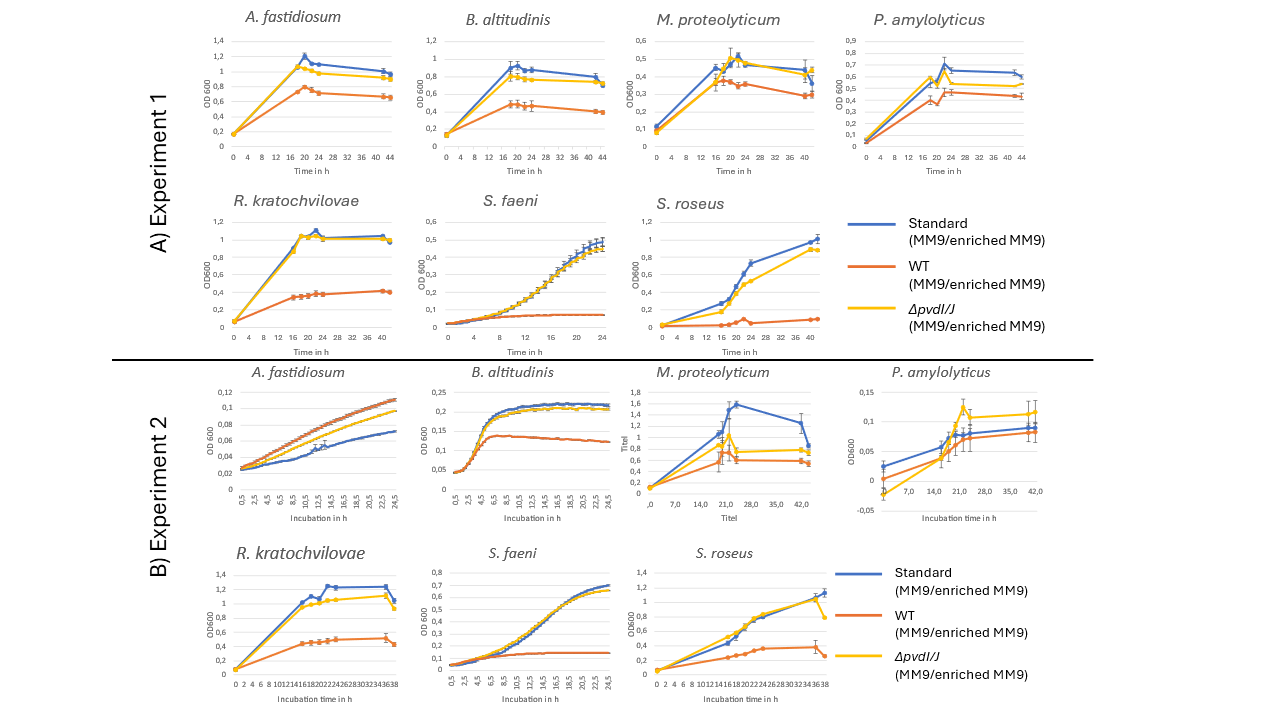


**Figure S15: Growth curves of SynCom members in presence or absence of pseudobactin** SynCom members were grown in MM9 or enriched MM9 in presence of sterile supernatant of P. koreensis WT (containing pseudobactin) and in presence of sterile supernatant of P. koreensis ΔpvdI/J mutant (no pseudobactin). Growth was observed by OD_600_ measurement at RT and 180 rpm shaking in an TECAN 2000 device. Experiments were performed in triplicates. For information on media (MM9 or enriched MM9) used for each strain see table. S1 and S2. A. humicola growth curve is not shown here, since it is shown in the article.


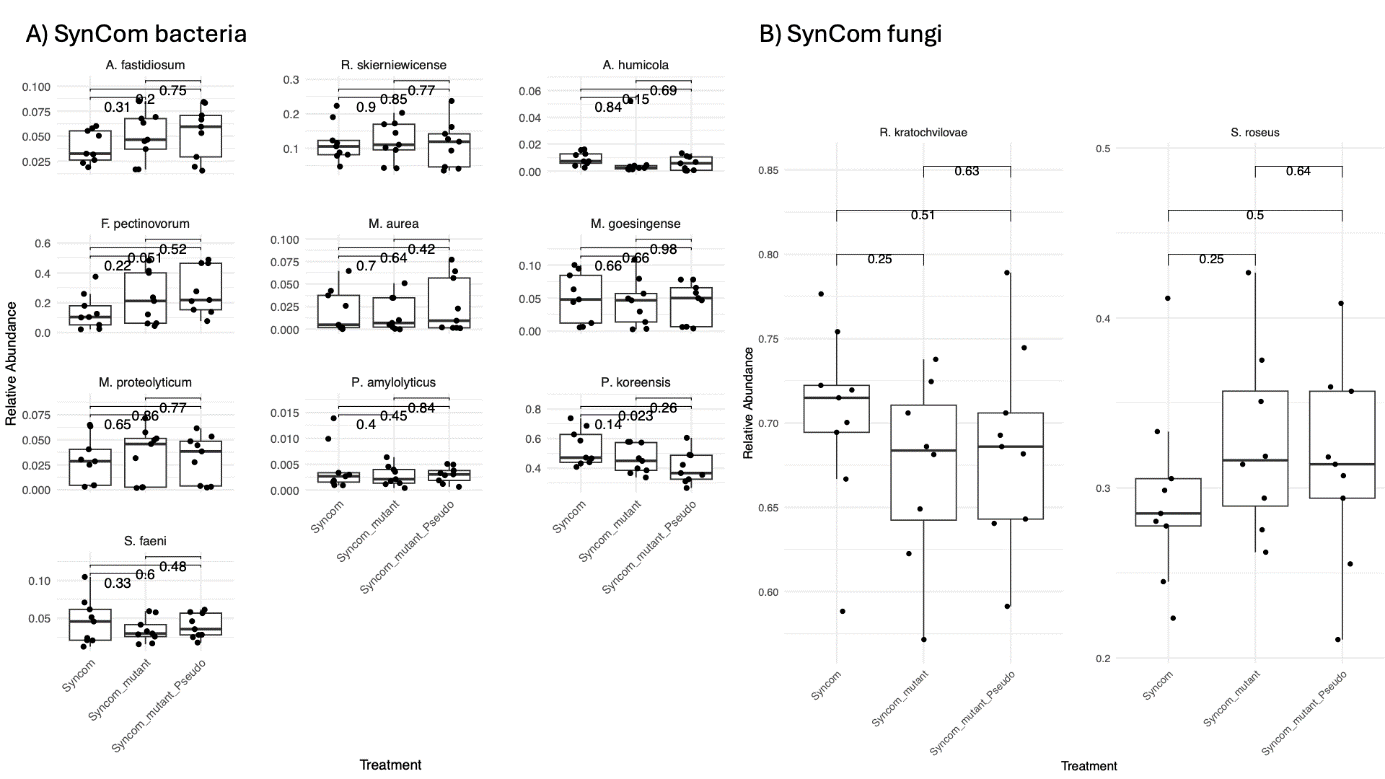


**Figure S16: T-test of each SynCom member for the experiment (Fig.6) after 5 days of incubation:** T-test was performed to see significant changes of the relative abundance of SynCom members grown on A. thaliana with SynCom WT, SynCom mutant and SynCom pseudobactin. *No data for D. hungarica, N. cavernae and B. altitudinis because the relative abundance was too low for the organisms.


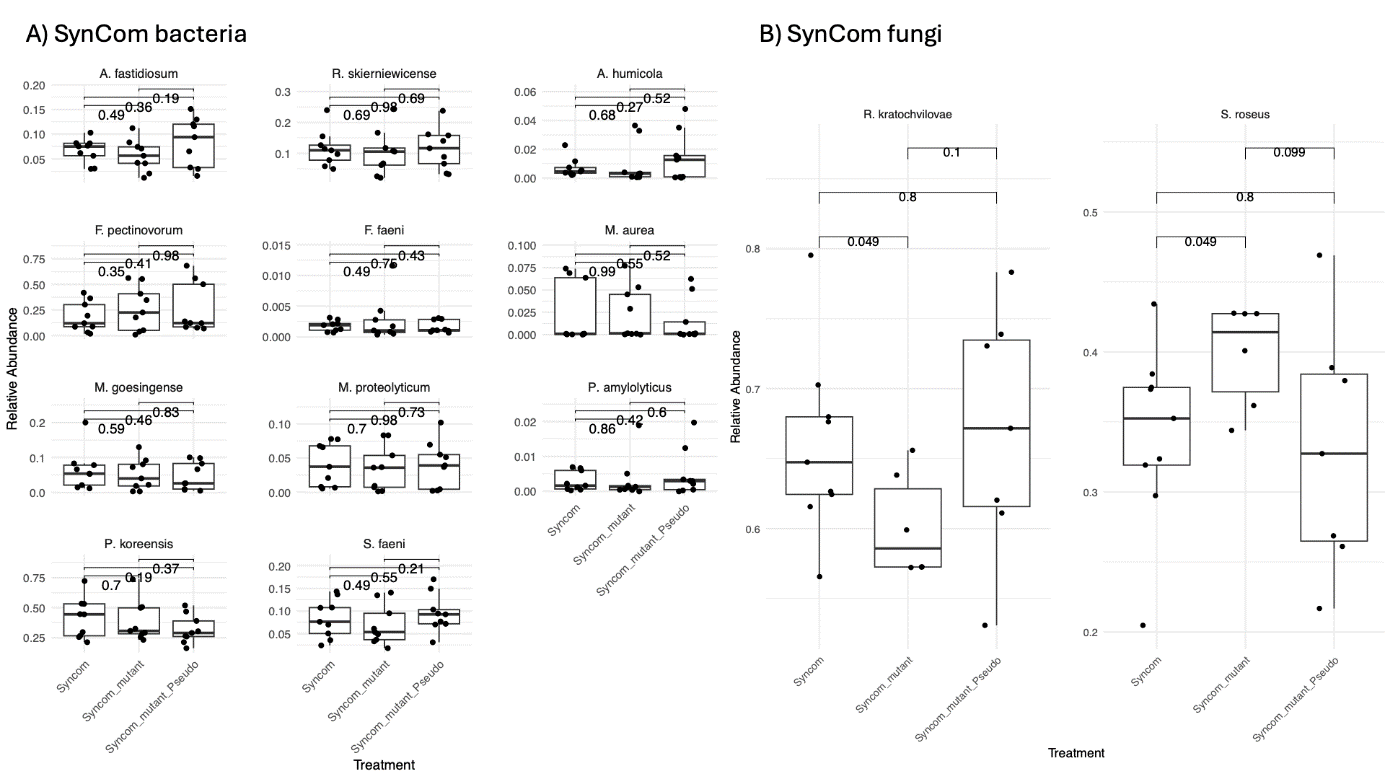


**Figure 17: T-test of each SynCom member for the experiment (Fig.6) after 9 days of incubation:** T-test was performed to see significant changes of the relative abundance of SynCom members grown on A. thaliana with SynCom WT, SynCom mutant and SynCom pseudobactin. *No data for D. hungarica, N. cavernae and B. altitudinis because the relative abundance was too low for the organisms.

**References**

*Huang et al., Huang W, Wilks A. A rapid seamless method for gene knockout in pseudomonas aeruginosa. BMC Microbiol. 2017;17:199 https://doi.org/10.1186/s12866-017-1112-5
